# The Effects of Menstrual Cycle Phase on Exercise Performance in Eumenorrheic Women: A Systematic Review and Meta-Analysis

**DOI:** 10.1007/s40279-020-01319-3

**Published:** 2020-07-13

**Authors:** Kelly Lee McNulty, Kirsty Jayne Elliott-Sale, Eimear Dolan, Paul Alan Swinton, Paul Ansdell, Stuart Goodall, Kevin Thomas, Kirsty Marie Hicks

**Affiliations:** 1 Department of Sport, Exercise and Rehabilitation, Faculty of Health and Life Sciences, Northumbria University, Newcastle-upon-Tyne, UK; 2 Department of Sport Science, Sport, Health and Performance Enhancement (SHAPE) Research Centre, Nottingham Trent University, Nottingham, UK; 3 Applied Physiology and Nutrition Research Group, Escola de Educação Física e Esporte, Faculdade de Medicina FMUSP, Universidade de São Paulo, São Paulo, Brazil; 4 School of Health Sciences, Robert Gordon University, Aberdeen, Scotland, UK

## Abstract

**Background:**

Concentrations of endogenous sex hormones fluctuate across the menstrual cycle (MC), which could have implications for exercise performance in women. At present, data are conflicting, with no consensus on whether exercise performance is affected by MC phase.

**Objective:**

To determine the effects of the MC on exercise performance and provide evidence-based, practical, performance recommendations to eumenorrheic women.

**Methods:**

This review followed the Preferred Reporting Items for Systematic Reviews and Meta-Analyses (PRISMA) guidelines. Four databases were searched for published experimental studies that investigated the effects of the MC on exercise performance, which included at least one outcome measure taken in two or more defined MC phases. All data were meta-analysed using multilevel models grounded in Bayesian principles. The initial meta-analysis pooled pairwise effect sizes comparing exercise performance during the early follicular phase with all other phases (late follicular, ovulation, early luteal, mid-luteal and late luteal) amalgamated. A more comprehensive analysis was then conducted, comparing exercise performance between all phases with direct and indirect pairwise effect sizes through a network meta-analysis. Results from the network meta-analysis were summarised by calculating the Surface Under the Cumulative Ranking curve (SUCRA). Study quality was assessed using a modified Downs and Black checklist and a strategy based on the recommendations of the Grading of Recommendations Assessment Development and Evaluation (GRADE) working group.

**Results:**

Of the 78 included studies, data from 51 studies were eligible for inclusion in the initial pairwise meta-analysis. The three-level hierarchical model indicated a trivial effect for both endurance- and strength-based outcomes, with reduced exercise performance observed in the early follicular phase of the MC, based on the median pooled effect size (ES_0.5_ = − 0.06 [95% credible interval (CrI): − 0.16 to 0.04]). Seventy-three studies had enough data to be included in the network meta-analysis. The largest effect was identified between the early follicular and the late follicular phases of the MC (ES_0.5_ = − 0.14 [95% CrI: − 0.26 to − 0.03]). The lowest SUCRA value, which represents the likelihood that exercise performance is poor, or among the poorest, relative to other MC phases, was obtained for the early follicular phase (30%), with values for all other phases ranging between 53 and 55%. The quality of evidence for this review was classified as “low” (42%).

**Conclusion:**

The results from this systematic review and meta-analysis indicate that exercise performance might be trivially reduced during the early follicular phase of the MC, compared to all other phases. Due to the trivial effect size, the large between-study variation and the number of poor-quality studies included in this review, general guidelines on exercise performance across the MC cannot be formed; rather, it is recommended that a personalised approach should be taken based on each individual's response to exercise performance across the MC.

**Electronic supplementary material:**

The online version of this article (10.1007/s40279-020-01319-3) contains supplementary material, which is available to authorized users.

## Key Points

**Table:** 

In women, exercise performance might be reduced by a trivial amount during the early follicular phase of the menstrual cycle when compared with other phases. However, large between-study variance was identified, indicating that research design, participant characteristics and choice of outcome measure might influence any group-level effect.
Practically, the current evidence does not warrant general guidance on modulating exercise across the menstrual cycle. As such, we recommend that a personalised approach should be taken based on each individual's response to exercise performance across the menstrual cycle.
The quality of evidence for this review was mostly classified as “low” quality, which can be attributed to a range of methodological issues. Future studies need to improve methodological quality and limit confounders to facilitate a deeper understanding of the effects of the menstrual cycle on exercise performance.

## Background

Over the last three decades, there has been a rise in the number of women participating in exercise, from physical activity to elite sport, attributable to the increasing development of, and investment in, women’s professional sport [1–4]. Specifically, the percentage of women competing at the Olympic Games has increased from 26% in Seoul in 1988 to 45% in Rio de Janeiro in 2016 [5]. Furthermore, Tokyo 2021 is set to be the most sex-balanced Games in history, with the same number of medals available for men and women, which is projected to see women participation in the Games rise to 49% [5]. Performance-based research in women has not kept pace with the exponential rise in participation [6, 7]. Indeed, it would be naive to assume that all research in men can be directly applied to women, given the anatomical, physiological and endocrinological differences between the sexes [4, 8–10]. As such, sportswomen will benefit from sex-specific research and guidelines, which consider the effects of women’s physiology, such as the menstrual cycle (MC), on performance [8, 11].

The MC is an important biological rhythm, whereby large cyclic fluctuations in endogenous sex hormones, such as oestrogen and progesterone, are observed [12–14]. The fairly predictable (and measurable) fluctuations in oestrogen and progesterone across the MC create significantly different transient hormonal profiles, which are used to differentiate between MC phases [15, 16]. As such, the MC is commonly divided into three phases, (1) the early follicular phase, characterised by low oestrogen and progesterone, (2) the ovulatory phase, characterised by high oestrogen and low progesterone, and (3) the mid-luteal phase, characterised by high oestrogen and progesterone [17]. Although the primary function of these hormones is to support reproduction, research has highlighted that the changing concentrations of oestrogen and progesterone across the MC also exert a myriad of diverse and complex effects on multiple physiological systems, including cardiovascular, respiratory, metabolic and neuromuscular parameters [12, 18, 19], which could have subsequent implications for exercise performance [15, 20–23].

There are a range of suggested mechanisms by which the cyclical fluctuations in oestrogen and progesterone across the MC might affect performance. Specifically, oestrogen is thought to have an anabolic effect on skeletal muscle [24, 25] and has been shown to play a role in substrate metabolism changes through increased muscle glycogen storage and increased fat utilisation [26]. Additionally, progesterone is thought to have anti-oestrogenic effects [21]. As such, it is plausible that changes in exercise performance might be observed due to the different hormonal profiles across the MC [15, 20–23]. To date, the effects of fluctuations in oestrogen and progesterone across the MC on exercise performance are conflicting, with studies reporting improved performance outcomes during the early follicular [27–29], ovulatory [30] and mid-luteal [31, 32] phases; whereas, others have shown no changes in exercise performance between MC phases [33–39]. Therefore, it is evident that a consensus is yet to be reached regarding the effects of the MC on exercise performance. Subsequently, no evidence-based guidelines for managing exercise performance across the MC currently exist for either exercising women, nor for practitioners working with elite sportswomen.

Given the recent increase in the number of women participating in exercise and the lack of consensus regarding the effects of the MC on exercise performance, there is a growing need to determine the effects of the fluctuations in oestrogen and progesterone across the MC on exercise performance. To our knowledge, this is the first meta-analysis to critically examine existing studies investigating changes in exercise performance across the MC, in eumenorrheic women. Additionally, this review is the first of its kind to appraise the quality of previous studies using robust assurance tools. The information provided by this meta-analysis can be used to inform practical recommendations for athletes, practitioners and researchers interested in managing exercise performance across the MC.

## Methods

This review conforms to the PRISMA statement guidelines (see Electronic Supplementary Material Appendix S1) [40].

### Study Inclusion and Exclusion Criteria

Consideration of Population, Intervention, Comparator, Outcomes and Study design (PICOS) was used to determine the parameters within which the review was conducted:

#### Population

Participants included healthy women who were (a) between the ages of 18 and 40 years; (b) eumenorrheic; (c) not taking any hormonal contraceptives or medication known to affect the hypothalamic–pituitary–ovarian (HPO) axis; (d) free from any menstrual-related dysfunctions (such as, amenorrhea) or any other conditions (e.g., pregnancy, eating disorders or disordered eating) known to affect the HPO axis; and (e) free from any injury that would affect participation. No restrictions were placed on activity level or training status.

#### Intervention

No specific intervention was investigated, but all participants were required to have a normal MC, defined as having a minimum of nine cycles per calendar year and a MC that ranged between 21 and 35 days in length.

#### Comparator

**Fig. 1:**
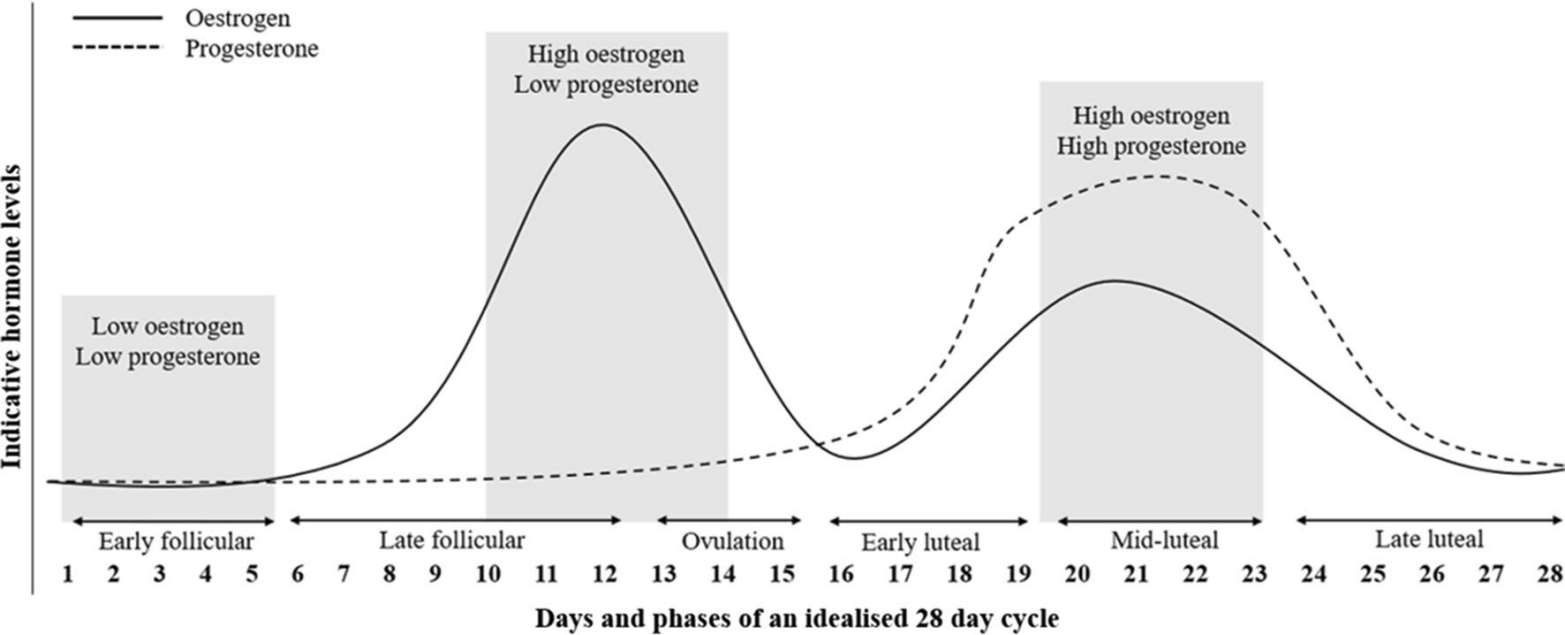
Schematic displaying the hormonal fluctuations across an idealised 28-day menstrual cycle, with ovulation occurring on day 14 Adapted from Pitchers and Elliott-Sale [8]

Comparisons were made between the early follicular phase (acting as a ‘control’ phase) of the MC and all other MC phases, in line with the following predetermined MC phase classification as shown in Fig. 1: early follicular (days 1–5), late follicular (days 6–12), ovulation (days 13–15), early luteal (days 16–19), mid-luteal (days 20– 23) and late luteal (days 24-28).

#### Outcomes

The primary outcome was exercise test performance. For the purposes of this review, exercise test performance was defined as total work done, time to completion, time to exhaustion, mean, peak and ratio outputs, rate of force production and decline, and indices of fatigue. Although maximum oxygen uptake (maximal [$\dot{V}$O_2max_] or peak [$\dot{V}$O_2peak_]) is not a performance test, this physiology-based outcome was included as it can be used as an indicator of performance. A full list of considered outcomes can be found in Electronic Supplementary Material Appendix S2. Performance outcome data were allocated into broad categories to allow for subgroup analysis; namely endurance (power and capacity) and strength (maximal expression of force and rate of force development). All exercise outcomes were extracted, and effect size duplication of multiple outcomes from the same test accounted for within the statistical analysis, as described below.

#### Study Design

Experimental studies were considered for analysis if they met the following inclusion criteria: (a) published, in full, in a peer-reviewed journal, (b) had the primary or secondary objective of assessing changes in exercise performance across the MC, (c) included within-group comparisons and (d) outcome measure(s) were taken in two or more defined MC phases. As such, case studies, review articles, study protocol papers and conference abstracts were excluded. Moreover, only full texts that were published in English or had an existing translation were retrieved and examined. There was no limit on date of publication.

### Search Strategy for Identification of Studies

A systematic electronic literature search was conducted by KLM to identify all relevant articles using four online databases (PubMed, CENTRAL, SPORTDiscus and ProQuest). The searches were performed using medical subject headings terms, free-text and thesaurus terms, as well as, keywords from existing relevant papers [15, 20–23]. The following search terms and their combinations were used: (‘menstrual cycle’, OR ‘menstrual phase’, OR ‘follicular phase’, OR ‘luteal phase’) AND (‘strength’, OR ‘power’, OR ‘torque’, OR ‘force’, OR ‘neuromuscular’, OR ‘max* voluntary contraction’, OR ‘isometric’, OR ‘isokinetic’, OR ‘skeletal muscle’ OR ‘muscular performance’, OR ‘aerobic’, OR ‘aerobic power’, OR ‘aerobic capacity’, OR ‘endurance’, OR ‘endurance power’, OR ‘endurance capacity’, OR ‘anaerobic’, OR ‘anaerobic power’, OR ‘anaerobic capacity’, OR ‘athletic performance’, OR ‘sports performance’). An example of a full electronic search for one database (PubMed: 14/01/2019) is presented in Electronic Supplementary Material Appendix S3. Databases were searched from inception until February 2019. The reference lists of obtained relevant articles and review articles were hand-searched to identify any further studies and were added in manually. Following the same search criteria and strategy, an updated electronic and manual hand-search for relevant literature was subsequently conducted in April 2020 to identify any further articles published between February 2019 and April 2020.

### Data Selection, Extraction and Study Quality Assessment

#### Selection of Studies

Three reviewers (KLM, KMH and KES) independently reviewed the titles, abstracts and full-text paper of the identified articles for inclusion and any duplicates were removed, using Covidence systematic review software (v1251, Veritas Health Innovation, Australia). All searches followed a two-phase screening strategy. Phase one assessed the eligibility of the title and abstract of every manuscript generated from the electronic searches and hand-searching against the predetermined inclusion and exclusion criteria. Studies that either clearly did not meet the inclusion criteria or met at least one exclusion criterion were excluded at this phase. In phase two, the full-text paper was retrieved for the articles identified in stage one and assessed against the predetermined inclusion and exclusion criteria. Any conflicts between the reviewers relating to study eligibility were resolved in consensus meetings (KLM, KMH and KES).

#### Data Extraction and Management

Data extraction was conducted by one reviewer (KLM), using a pre-piloted data extraction form, and independently verified by two members of the review team (KMH and KES). Any discrepancies were resolved by reviewing the original article and consensus achieved by discussion during consensus meetings (KLM, KMH and KES), or, if needed, in consultation with a fourth reviewer (ED). When data were presented in graphical and not in numerical format, DigitizeIt software (v2.3, DigitizeIt, Germany) was used to convert the relevant data. Further, where data were incomplete, authors were contacted to obtain the relevant information. Authors were given 4 weeks to respond; if the authors failed to respond after this date, the papers were excluded if no relevant data could be extracted from the published version of the paper.

#### Quality Assessment of Included Studies

Study quality was assessed by one reviewer (KLM) and independently verified by two members of the review team (KMH and KES), using a strategy based on the recommendations of the Grading of Recommendations Assessment Development and Evaluation (GRADE) working group [41]. This strategy considers quality of evidence for any one outcome based on five domains, namely risk of bias, indirectness, inconsistency, imprecision or evidence of publication bias. Both risk of bias and indirectness were initially conducted at the individual study level, with mode ratings used to describe whole outcomes. The initial appraisal tool used was based on the Downs and Black checklist for measuring study quality [42] and was specifically modified for use in this review (see Electronic Supplementary Material Appendix S4). The modified Downs and Black checklist comprised 15 outcomes, from five domains: (1) reporting; (2) external validity; (3) internal validity—bias; (4) internal validity—confounding; and (5) power. A maximum attainable score of 16 could be awarded, whereby study quality was categorised as follows: “high” (14–16); “moderate” (10–13); “low” (6–9); or “very low” (0–5). The results of the Downs and Black assessment were used to assign an a priori quality rating to each study. This a priori rating was then either maintained, or downgraded a level, based on the response to two questions that were considered key to the *directness* of these research studies: Q.1) was the MC phase confirmed using blood samples? If the authors reported using blood samples to confirm MC phase, the a priori rating was maintained and if not, the study was downgraded a level (*e.g.,* a study that started out as “high” in quality, but did not confirm MC phase using a blood sample, drops to “moderate” in quality); and Q.2) was the MC phase confirmed using urinary ovulation detection kits? If the authors reported the use of an urinary ovulation detection kit to identify MC phase, the Q.1 rating was maintained; if not, the study was downgraded a level (as such, the maximum rating for any study that does not use serum analysis or urinary ovulation detection kits to identify and verify MC phase is “low”). The inclusion of these specific questions was based on the methodological conclusions made in previous studies [10, 17]. Consistency was ascertained using the meta-analysis results and was based on visual inspection of effect size estimates, whether or not confidence intervals overlapped, and on statistical tests for heterogeneity. Precision was judged based on the number of outcomes available (with outcomes based on < 5 data points downgraded) and on visual analysis of the width of the confidence intervals. Publication bias was assessed using Egger’s test along with visual inspection of funnel plots. Overall, this procedure allowed the final quality of evidence for each outcome to be categorised as either “high”, “moderate”, “low” or “very low” in quality. This quality appraisal was not used to exclude any study, although a sensitivity analysis was conducted using only those individual studies deemed to be of “high” or “moderate” quality, based on the risk of bias and directness assessments. Any differences between the reviewers were resolved by discussion during consensus meetings (KLM, KMH and KES), or, if needed, in consultation with a fourth reviewer (ED).

### Data Synthesis

Data were extracted from studies comprising both between- and within-group designs. Pairwise effect sizes were calculated by dividing mean differences by pooled standard deviations. At the study level, variance of effect sizes was calculated according to standard distributional assumptions [43]. All meta-analyses were conducted within a Bayesian framework enabling the results to be interpreted more intuitively compared to a standard frequentist approach through use of subjective probabilities [44]. With a Bayesian framework, dichotomous interpretations of the results of a meta-analysis with regards to the presence or absence of an effect (e.g. with *p* values) can be avoided, and greater emphasis placed on describing the most likely values for the average effect and addressing practical questions such as, the probability the average effect is beyond a certain threshold [44]. The Bayesian framework is also particularly suited to hierarchical models and sharing information within and across studies to improve estimates [44]. In the present meta-analysis, three-level hierarchical models were conducted to account for covariance in multiple outcomes presented in the same study [45]. For the initial analysis, individual effect sizes were calculated by comparing exercise performance in the early follicular phase (acting as a ‘control’ phase) with all other phases of the MC (late follicular, ovulation, early luteal, mid-luteal and late luteal). Meta-regression was performed to assess whether the pooled effect size estimate was influenced by testing category (endurance or strength outcomes). Where no evidence of a difference was identified, the model was re-run combining both categories of outcomes to increase data to better estimate model parameters. Given the expectation of relatively small effect sizes, an a priori threshold of ± 2 was identified for outliers. Primary analyses were completed with outliers removed, but results were also presented from the full complement of studies as sensitivity analyses. A sensitivity analysis was also conducted on data obtained from studies categorised as “high” or “moderate” in quality. Assessment of publication bias was made using a multilevel extension of Egger’s test with effect sizes regressed on the inverse of standard errors [46]. Inferences from all analyses were performed on posterior samples generated by Markov Chain Monte Carlo with Bayesian 95% credible intervals (CrIs) constructed to enable probabilistic interpretations of parameter values. Interpretations were based on visual inspection of the posterior sample, the median value (ES_0.5_: 0.5 quantile) and 95% CrIs. Cohen's [47] standard threshold value of 0.8 was used to describe effect size as large, values between 0.5 and 0.8 as medium, values between 0.2 and 0.5 as small, and values between 0 and 0.2 as trivial.

Subsequent to this initial analysis, a network meta-analysis approach was used to compare exercise performance measured across all MC phases (early follicular, late follicular, ovulation, early luteal, mid-luteal and late luteal) with each other. Network meta-analyses are becoming increasingly common in evidence synthesis and are most commonly used to compare multiple experimental treatments where individual studies are unlikely to directly compare all relevant treatments [48]. The technique calculates pairwise effect sizes from studies comparing two treatments (direct evidence), and generates indirect evidence comparing other treatments through a common comparator [48]. The technique was adopted in the present review to supplement the initial pairwise meta-analysis and synthesise additional data comparing exercise performance using different combinations of MC phases. Study-specific treatment effects were drawn from multivariate normal distributions with up to five arms included. To test the consistency assumption of the network meta-analysis, the fit of the base-case model was compared to that of the inconsistency model. To summarise potential differences in exercise performance outcomes across all MC phases, results from the network meta-analysis were used to calculate the Surface Under the Cumulative Ranking curve (SUCRA; [49]). For each MC phase, a SUCRA value expressed as a percentage was calculated representing the likelihood that exercise performance was maximised or near maximised relative to other MC phases. More formally, the SUCRA value can be interpreted as the average proportion of phases where exercise performance is lower than the phase considered, with the mean SUCRA value equal to 50% [50]. Analyses were performed using the R packages R2WinBUGS [51] and brms [52]. Convergence of parameter estimates was checked with Gelman–Rubin R-hat values [53].

## Results

### Literature Search

**Fig. 2:**
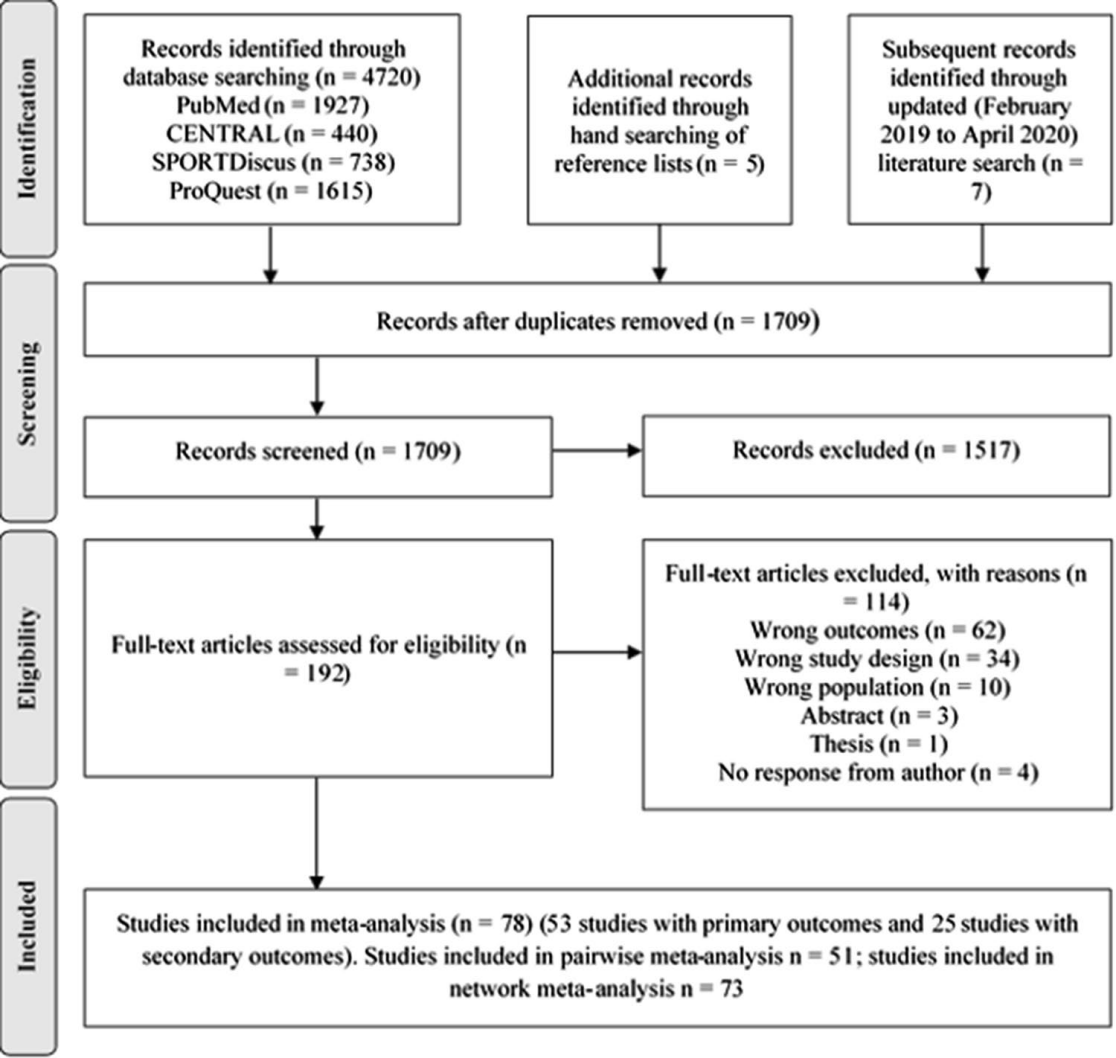
Preferred Reporting Items for Systematic Reviews and Meta-Analyses (PRISMA) guidelines flow chart for literature search and study selection

The literature search and selection of studies are presented in Fig. 2.

### Study Characteristics

In total, 78 studies [19, 27–39, 54–117] with a total of 1193 participants were included in the review. Details of the included studies are shown in Electronic Supplementary Material Appendix S5.

### Methodological Quality

#### Quality Assessment of Included Studies

**Fig. 3:**
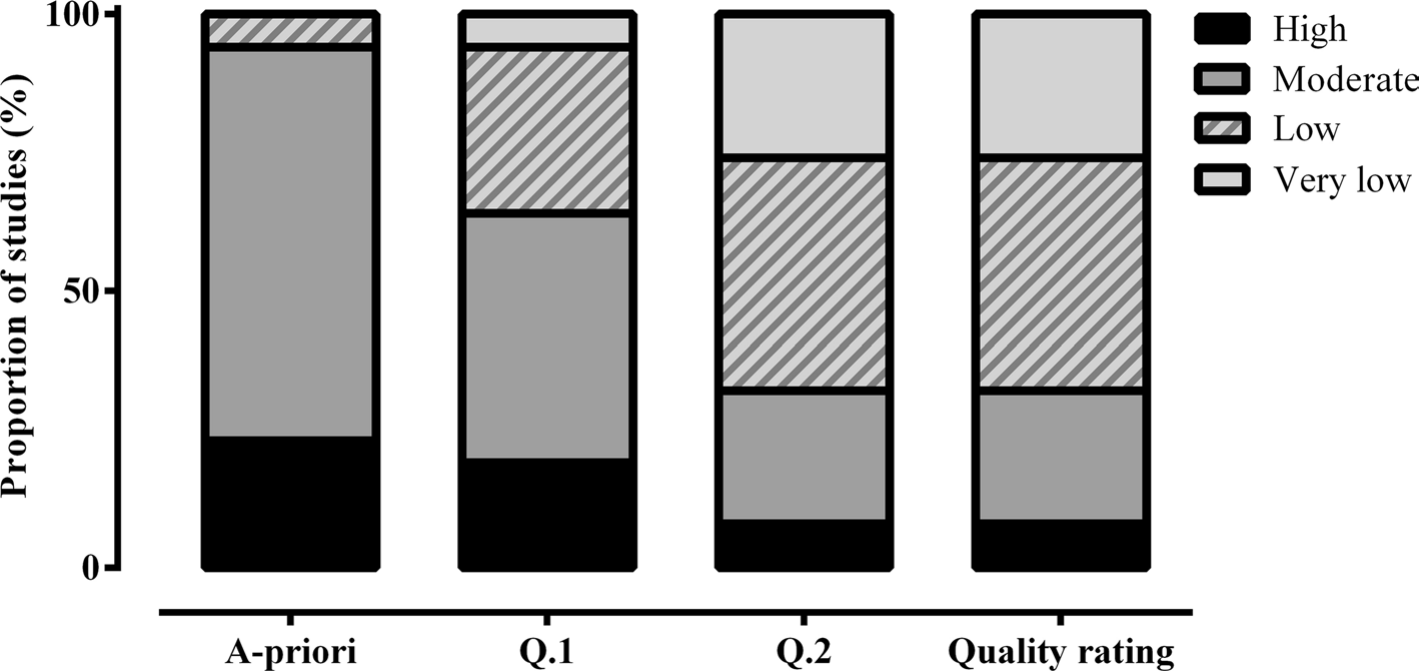
Quality rating of outcomes from all included studies (*n* = 78). Each bar represents the proportion of studies assigned a “high,” “moderate,” “low,” or “very low” quality rating. The *x*-axis represents the different stages of the quality appraisal process, with question one (Q. 1) and question two (Q. 2) indicating the questions asked to determine menstrual cycle phase identification and verification in each study, with the final bar representing the proportion of studies assigned to each quality rating category

All quality classifications are presented in Fig. 3. Analysis of quality based on the entire evidence base (*n* = 78) was ascertained at the individual study level, and according to the Downs and Black checklist, as well as the additional questions regarding MC phase confirmation. The quality of the evidence from the 78 studies included in this review was primarily classified as “low” in quality (8% “high”; 24% “moderate”; 42% “low”; 26% “very low”; Fig. 3) such that, “our confidence in the effect estimate is limited: the true effect might be substantially different from the estimate of the effect” [118]. In particular, 71% of studies were initially allocated an a priori rating of “moderate” quality; however, following the application of questions pertaining to MC phase identification and verification, only 24% of these studies were allocated a final rating of “moderate” quality.

#### Menstrual Cycle Phase Identification and Verification

In the 78 included studies, an array of methods was used to identify MC phase: (1) a combination of methods (*e.g.* counting of days, basal body temperature [BBT], assessment of menstrual symptoms, MC history and serial follicular scanning] without urinary ovulation detection kits (45%); (2) a combination of methods (e.g. counting of days, BBT, MC history, assessment of menstrual symptoms and urine ovulation detection kits) with urinary ovulation detection kits (31%); (3) counting of days (10%); (4) MC history (4%); (5) BBT (4%); and (vi) urinary ovulation detection kits (1%). In addition, some studies (5%) did not provide any information on how MC phases were identified. In relation to MC phase verification, out of the 78 studies included in the review, the majority of studies (59%) retrospectively verified MC phase using serum oestrogen and progesterone, a small number of studies retrospectively verified MC phase using saliva (4%) or urine (2%) oestrogen and progesterone, and the remaining studies provided no information on how they verified the identified MC phase (35%).

### Outcomes

#### Analysis 1: Pairwise Meta-Analysis

**Fig. 4:**
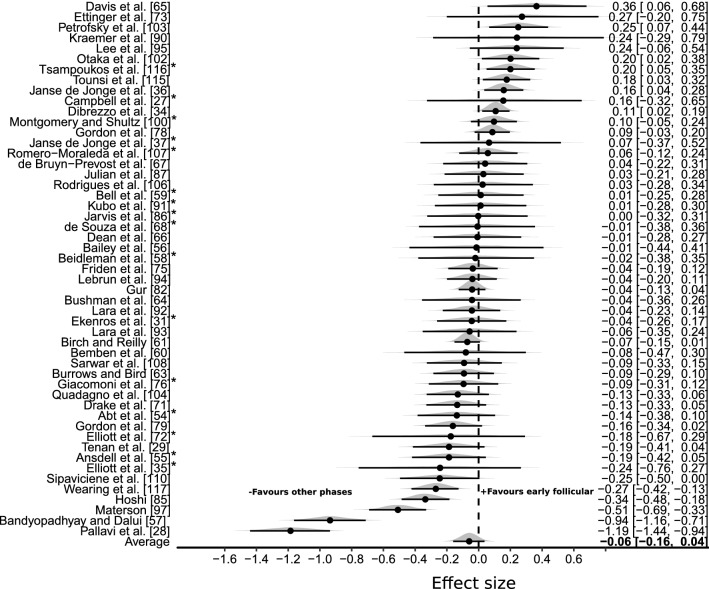
Bayesian Forest Plot of multilevel meta-analysis comparing performance measured during the early follicular phase with all other menstrual cycle phases. The study-specific intervals represent individual effect size estimates and sampling error. The circle represents the pooled estimate generated with Bayesian inference along with the 95% credible interval (95% CrI). Negative values favour all other menstrual cycle phases (late follicular, ovulation, early luteal, mid-luteal and late luteal) compared to the early follicular phase. High and moderate quality studies are indicated with an asterisk (*)

The initial meta-analysis comprised pooling of pairwise effect sizes comparing exercise performance during the early follicular phase of the MC with all other MC phases (late follicular, ovulation, early luteal, mid-luteal and late luteal). From the 78 studies that were eligible for the systematic review, 51 studies [19, 27–29, 31, 34–37, 54–60, 62–67, 70–72, 74, 75, 77, 78, 81, 84–86, 89–94, 96, 99, 101–103, 105–107, 109, 114–116] included assessment of exercise performance during the early follicular phase of the MC and included all other data required for calculations. The 51 studies (mode quality rating = “low”; 8% “high”; 24% “moderate”; 37% “low”; 31% “very low”) generated 362 pairwise effect sizes (240 strength and 122 endurance) with an average of four outcomes per study and a range from 1 to 12 outcomes. Data were obtained from 709 participants with studies comprising a mean participant size of 14 (range *n* = 5–100). A total of nine outliers were identified (seven studies with effect sizes less than −2 [favoring the “other MC phases”] and two studies with effect sizes greater than +2 [favoring the early follicular phase]) and subsequently removed from the analysis. The three-level hierarchical model indicated a trivial effect with reduced performance obtained in the early follicular phase of the MC, based on the median pooled effect size (ES_0.5_ = − 0.06 [95% CrI: − 0.16 to 0.04]; Fig. 4). Large between-study variance was identified ($\tau$_0.5_ = 0.26 [0.18–0.38]) and interclass correlation coefficient estimates close to zero indicated little within-study correlation between outcomes. Pooling of strength and endurance outcomes was conducted as no evidence was obtained that indicated a differential effect between these performance categories (ES_0.5/Endurance-Strength_ = − 0.01 [95% CrI: − 0.18 to 0.16]). Posterior estimates of the pooled effect size indicated close to zero probability of a small effect either in favour of the early follicular phase or all other MC phases (*d* ≥ 0.2; *p* ≤ 0.001). Egger’s regression test provided no evidence of publication bias (Egger_0.5_ = –0.01 [95% CrI: − 0.09 to 0.08]). Inclusion of outliers within the model had minimal influence on the average effect size (ES_0.5_ = − 0.08 [95% CrI: − 0.21 to 0.05]) and between-study variance ($\tau$_0.5_ = 0.30 [95% CrI: 0.23–0.39]). A sensitivity analysis was completed with data obtained from studies classified as either “high” or “moderate” in quality (16 studies compromising 38 strength effect sizes and 12 endurance effect sizes from 169 participants [19, 27, 31, 35, 37, 54, 57, 58, 67, 71, 75, 85, 90, 99, 106, 115]). Compared to the primary analysis, the reduced data set resulted in a relatively symmetric credible interval around the zero value (ES_0.5_ = − 0.01 [95% CrI: − 0.11 to 0.08]).

#### Analysis 2: Network Meta-Analysis

**Fig. 5:**
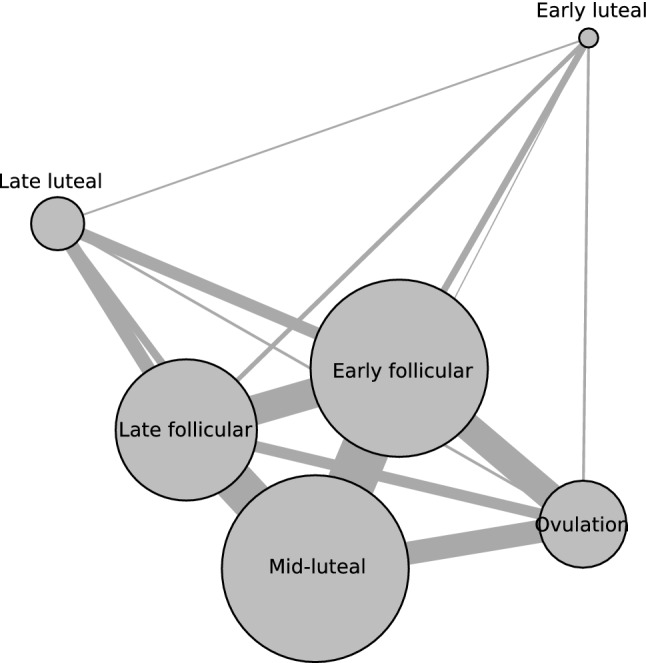
Network diagram illustrating the pairwise effect sizes calculated across the six menstrual cycle phases (early follicular, late follicular, ovulation, early luteal, mid-luteal and late luteal). The analysis included direct and indirect pairwise effect sizes from 73 studies. The relative size of nodes and relative thickness of connecting lines illustrate the frequency of outcomes measured in a given menstrual cycle phase and the number of direct comparisons between two phases, respectively

Figure 5 shows a network diagram illustrating the pairwise effect sizes calculated across the six MC phases (early follicular, late follicular, ovulation, early luteal, mid-luteal and late luteal). Seventy-three studies (mode quality rating = “low”; 7% “high”; 26% “moderate”; 42% “low”; 25% “very low”) included enough data to be included in the network meta-analysis [19, 27–29, 31, 33–39, 54–68, 70–72, 74, 75, 77–117]. A total of 220 performance outcomes were included across 954 participants, with the number of comparisons across MC phases equal to: comparison between two phases = 87; comparison between three phases = 93; comparison between four phases = 27; comparison between five phases = 10; and comparison between six phases = 3. The most frequent comparisons made were between the early follicular and mid-luteal phase of the MC (21% of comparisons), followed by the late follicular and mid-luteal phases of the MC (18% of comparisons). Pairwise estimates including the early follicular phase as a reference are presented in Table 1. with negative median pooled effect sizes (“other MC phases”) obtained for all comparisons and the largest effect identified between the early follicular and the late follicular phase of the MC (ES_0.5_ = − 0.14 [95% CrI: − 0.26 to − 0.03]). The lowest SUCRA value was obtained for the early follicular phase (30%) with all other MC phase values ranging between 53 and 55%.

**Table 1:** Summary of network meta-analysis results from 73 studies using the early follicular phase as a reference

Comparison to early follicular phase	Effect size [95% CrI]	SUCRA (%)
Early follicular	−	30
Late follicular	− 0.14 [− 0.26 to − 0.03]	54
Ovulation	− 0.07 [− 0.15 to 0.07]	55
Early luteal	− 0.07 [− 0.19 to 0.16]	54
Mid-luteal	− 0.04 [− 0.11 to 0.08]	55
Late luteal	− 0.01 [− 0.18 to 0.17]	53

Negative values for effect sizes favour all other menstrual cycle phases (late follicular, ovulation, early luteal, mid-luteal and late luteal) compared to the early follicular phase

*SUCRA* the surface under the cumulative ranking curve, *CrI* credible intervals

## Discussion

The aim of this review was to examine if MC phase affects exercise performance in eumenorrheic women. The results indicate that on average, exercise performance might be trivially reduced during the early follicular phase of the MC when compared with all other MC phases. Performance was consistent between all other MC phases. In addition to the estimated trivial average effect, results from the meta-analysis models showed relatively large between-study variance indicating that research design, participant characteristics and type of performance measured might influence any effect. Furthermore, most studies that were included in this meta-analysis were classified as “low” in quality, and as such, the confidence in the evidence reported in this meta-analysis is also low, and should be interpreted with caution. Due to the trivial effect size, the large between-study variation and the number of poor-quality studies included in this review, general guidelines on exercise performance across the MC cannot be formed; rather, it is recommended that a personalised approach should be taken based on each individual's response to exercise performance across the MC.

There are a range of suggested mechanisms by which the lower levels of oestrogen and progesterone seen in the early follicular phase of the MC might negatively affect the exercise performance. Although a detailed mechanistic review is beyond the scope of this review, the following points can be noted. First, oestrogen is known for its anabolic effects [24, 25], as well as its role in regulating substrate metabolism through increasing glycogen uptake and sparing glycogen stores. Additionally, it has been shown to have antioxidant and membrane stabiliser properties, which might offer protection against exercise-induced muscle damage and reduce inflammatory responses [26]. Further, oestrogen is thought to have neuroexcitatory effects, whereby it reduces inhibition and increases voluntary activation [19]. Therefore, when oestrogen rises during the late follicular and ovulatory phases and remains elevated in the mid-luteal phase, it is plausible that this might affect muscular performance [24, 25] or maximal and submaximal intensity exercise performance [26]. Moreover, progesterone is thought to have anti-oestrogenic effects [21]; therefore, it could be speculated that the beneficial performance effects of oestrogen are likely to be greater in the late follicular and ovulatory phases when oestrogen is high without the interference of progesterone, compared to the mid-luteal phase when *both* oestrogen and progesterone are high. This speculation is supported by the finding presented here that the biggest difference in performance was between the early follicular and late follicular phases of the MC. However, the average effect calculated was trivial and there was considerable overlap between each of the pairwise comparisons with the early follicular phase. Whilst the current meta-analysis cannot identify the mechanisms responsible, it does indicate that, on average, exercise performance might be reduced by a trivial amount in the early follicular phase of the MC compared with all other phases. Interestingly, our sister meta-analysis, on the effects of oral contraceptives (OCs) on exercise performance, showed that, compared with eumenorrheic women, OC users have on average slightly inferior exercise performance [119]. Oral contraceptive use results in significantly downregulated concentrations of endogenous oestrogen and progesterone when compared with the ovulatory and mid-luteal phases of the MC [71]. Indeed, the endogenous hormonal profile of OC users is comparable to the profile seen during the early follicular phase of the MC [71]. Both meta-analyses show slightly impaired, group-level, exercise performance when *both* oestrogen and progesterone are at their lowest, therefore collectively suggesting that exercise performance might be mediated by the concentration of endogenous ovarian hormones in some exercising women.

Within the literature to date, the most common comparison used when investigating the effects of the MC on performance was between the early follicular and mid-luteal phase. This is not surprising, as the difference in the hormonal milieu is typically at its greatest between these phases (early follicular when *both* oestrogen and progesterone are low, and mid-luteal when *both* oestrogen and progesterone are high) [17]. As such, if performance was altered by synergistic fluctuations in oestrogen and progesterone levels, the comparison between these two phases would maximise the chance of observing an effect. This bi-phasic comparison, however, ignores the late follicular and ovulatory phases of the MC, when oestrogen is high, and progesterone is low. The network analysis indicated that the largest difference in performance might be expected between the early follicular and the late follicular phases of the MC, when *both* oestrogen and progesterone are low and when oestrogen rises without a concurrent increase in progesterone. Therefore, the effects of oestrogen, without the interference of progesterone, might be overlooked if the late follicular or ovulatory phases are not included within the phase comparisons. Future studies should, therefore, consider multiple phase comparisons so that the effects of different ratios of oestrogen and progesterone can be explored. It should be noted, however, that the inclusion of multiple phase comparisons will result in more variability, and as such, more participants will be needed to conclude any potential effects.

Although this systematic review included 78 studies and 1193 women (range *n* = 5–100), there were very few studies classified as “moderate” or “high” in quality, which implies that the confidence in the evidence used in this meta-analysis should be low. Specifically, only 24% of studies were allocated a quality rating of “moderate”, and only 8% of studies were allocated a quality rating of “high”. Our quality assessment approach included consideration of the methods used to identify and verify the MC phase in the included studies, which is considered to be key to the trustworthiness of the results obtained (i.e. Q1. was the MC phase confirmed using blood samples; Q2. was the MC phase confirmed using urinary ovulation detection kits?). Across the included studies there was large variability in the methods used to identify and then verify MC phase, namely calendar-based counting, BBT, MC history questionnaires, urinary ovulation detection kits, and salivary, urinary and serum measurement of both oestrogen and progesterone. Calendar-based counting is an indirect method to identify MC phase, whereby the self-reported onset of menses is set as day one, and the phases are then established by counting days from this point [17]. This method, however, assumes that all participants with regular menstruation experience ovulatory cycles with a mid-cycle peak in oestrogen, which is not always the case [120, 121]. As such, the use of calendar-based counting methods in isolation is not recommended when accurate identification of MC phase is required [122]. Similarly, BBT is a widely used method for identifying ovulation, and the length of the follicular and luteal phases [17], but this method does not provide information regarding actual hormone concentrations [123], and temperature readings might also be influenced by a range of factors such as illness, stress, sleep patterns and medication [124]; hence BBT in isolation is not considered a reliable method for MC phase verification [17]. Studies using these aforementioned methods were downgraded on this basis. Indeed, very few studies used a combination of the recommended methods by Cable and Elliott [10] and Janse de Jonge et al. [17], which include the use of the calendar-based counting method in conjunction with urinary ovulation detection kits to assist in setting the timing of testing throughout the MC and to confirm the presence of an ovulatory cycle, followed by serum measurement of both oestrogen and progesterone levels to subsequently verify the phases of the MC. Given that the rationale for exploring the effect of the MC on performance is underpinned by changes in oestrogen and progesterone, it is essential that studies should accurately verify the acute changes in endogenous hormones during each phase of the MC to ensure that the intended phase is being examined. Overall, without blood analysis, it is unclear which hormone milieu is being investigated, thus making it difficult to draw accurate conclusions regarding changes in performance across the MC and to make direct comparisons between studies. These recommendations echo recent publications in the area of women’s physiology [10, 17], demonstrating an increasing awareness for the nuances of this type of research, and collectively provide researchers with ample tools to make methodological decisions for future investigations. To limit the influence of low quality papers on the analyses, a sensitivity analysis was conducted with data obtained from studies that were classified as either “moderate” or “high” in quality [19, 27, 31, 35, 37, 54, 57, 58, 67, 71, 75, 85, 90, 99, 106, 115]. Due to the limited amount of data available, only the pairwise meta-analysis comparing exercise performance during the early follicular phase of the MC with all other MC phases was conducted. The sensitivity analysis provided no evidence of any effect, with a relatively symmetric credible interval centred close to zero. Whilst studies that were allocated a higher quality rating were better able to identify and verify the MC phase, there was no association between study quality and average sample size. Given the reduced amount of data included within the sensitivity analysis and the low sample sizes, the result is consistent with the primary analyses and conclusion that if an average effect exists, it is likely to be trivial in magnitude.

The results from the meta-analysis models consistently showed large between-study variance, which might be attributable to several factors: (a) inconsistent research design, as shown by the network analysis that highlights the discrepancy in the number of phase comparisons made between studies; (b) poor methodological practices, as emphasised by the quality assessment, whereby the majority of studies included in the meta-analysis were classified as “low” (42%) in quality primarily due to inadequate MC phase identification and verification in many studies; (c) non-homogenous participant groups, as shown in Electronic Supplementary Material Appendix S5 participants in this meta-analysis ranged from sedentary, to healthy, to physically active to elite athletes; and (d) large variation in the type of performance outcome measured, as detailed in Electronic Supplementary Material Appendix S2. As such, the breadth of this research area, without the corresponding depth, makes it difficult to apply a meaningful, yet generalisable, interpretation of the current data.

## Conclusion

This is the first systematic review with meta-analysis to examine the effect of MC phase on exercise performance in eumenorrheic women. These data provide new information that exercise performance might on average be reduced by a trivial amount during the early follicular phase of the MC, compared with all other MC phases. The current meta-analysis also identified large between-study variance in the effect of the MC on exercise performance. This might have been influenced by a range of methodological factors and small participant numbers (average *n* = 14) as well as associated high sampling variance. Participant characteristics, such as training history, might also have contributed to the large between-study variance observed. From a practical perspective, as the effects tended to be trivial and variable between studies, the implications of these findings are likely to be so small as to be meaningless for most of the population. These trivial effects might, however, be of greater relevance to elite athletes, where the difference between winning and losing is marginal. Specifically, we recommend that practitioners working with elite sportswomen need to consider the MC and be aware of the potential times across the cycle whereby exercise performance might be reduced (early follicular phase) or enhanced (all other MC phases), but this approach should be tailored to, and informed by, the individual athlete. In the future, it would be interesting to identify which factors might cause some women to experience reduced performance during the early follicular phase of the MC when compared with all other MC phases, and identify strategies to monitor these effects. Therefore, future studies need to improve methodological quality (e.g., appropriate biochemical outcomes to confirm MC phase) and limit confounders to facilitate a deeper understanding of the effects of the MC on exercise performance in individuals.

## Electronic supplementary material

Below is the link to the electronic supplementary material.

Supplementary material 1 (DOCX 19 kb)

Supplementary material 2 (DOCX 37 kb)

Supplementary material 3 (DOCX 14 kb)

Supplementary material 4 (DOCX 16 kb)

Supplementary material 5 (DOCX 64 kb)

## Data Availability

Please contact the corresponding author for data requests.
