# Supplementary material for: The Effects of Menstrual Cycle Phase on Exercise Performance in Eumenorrheic Women: A Systematic Review and Meta-Analysis
Source: Sports Med. 2020 Jul 13;50(10):1813–27. doi: 10.1007/s40279-020-01319-3 (PMC7497427; doi:10.1007/s40279-020-01319-3)
Supplement: Supplementary file 2 — Supplementary material 2 (DOCX 37 kb) [file 40279_2020_1319_MOESM2_ESM.docx]

The Effects of Menstrual Cycle Phase on Exercise Performance in Eumenorrheic Women: A Systematic Review and Meta-Analysis. Sports Medicine. Corresponding author: Dr Kirsty Elliott-Sale, Sport Health and Performance Enhancement (SHAPE) Research Centre, Department of Sport Science, Nottingham Trent University, Nottingham, UK. Email: kirsty.elliottsale@ntu.ac.uk.

**Electronic Supplementary Material Appendix S2.** Full List of Considered Outcomes from the 78 Included Studies.

| Considered outcome(s) |
| --- |
| Strength |
| Hamstring:quadricep strength ratio at 60 and 180º s^–1^ [1]  Maximum voluntary contraction (N) with motor nerve stimulation [2] |
| Time to task failure (s) during fatiguing task involving sets of intermittent isometric contractions performed in the lower-body [2]  Peak isokinetic torque of the knee flexors and extensors at 1.05 and 3.14 rad.s^-1^ through 90º range of motion on an isokinetic dynamometer (Nm) [3]  Maximal voluntary isometric contraction of the knee flexors and extensors measured at 0 rad.s^-1^ and 60º of knee flexion with electrical stimulation (Nm) [3] |
| Rate of force production during a maximal voluntary isometric hamstring contraction (N.s^-1^) [4]  Time to 50% peak force during a maximal voluntary isometric hamstring contraction (ms) [4]  Maximal isometric lifting strength (MILS) performed at both knee and waist height (N) [5, 6]  Time to volitional fatigue at 45% MILS performed at both knee and waist height (s) [5, 6]  Maximal acceptable load (kg) [5] |
| Handgrip strength (N) [7, 8, 9] |
| Standing long jump performance x body mass (kg.m) [7] |
| Mean peak torque of knee flexors and extensors at 60, 180, 240º.s (Nm) [10]  Muscular endurance and work ratios of knee flexors and extensors at 240º.s [10]  Maximal torque produced during an isometric muscle action (Nm) [11]  Torque produced at a sub-maximal (20, 50 and 75%) isometric muscle action (Nm) [11]  Peak isokinetic muscle torque of knee extensors at 120º.s (Nm) [12, 13]  Handgrip strength (kg) [12, 13, 14, 15, 16, 17]  Peak length of hop during a one-leg hop test (cm) [12, 13] |
| Maximum voluntary isometric force of the first dorsal interosseous (N) [18, 19] |
| Maximal jump power from a multi-jump test (W.kg) [20]  Maximal jump height from a squat jump test (cm) [20] |
| Torque production (Nm) of the knee extensors and flexors at 60, 80, 120 and 240º.s [21] |
| Torque ratios (peak and total work) during concentric and eccentric hamstring and quadriceps testing at 60 and 180º.s [22]  Peak torque of quadricep and hamstring flexors and extensors at 120º.s (Nm) [23]  Hamstring:quadricep strength ratio [23]  Time to task failure during a sustained isometric fatiguing contraction at 25% of MVC (s) performed in the upper-body [24]  Back lift strength (kg) [15] |
| Isometric quadricep strength (N) at 60º with electrical stimulation [8]  Isokinetic strength of the quadricep flexors and extensors at 60˚.s^-1^ and 140º.s^-1^ (Nm) [8] |
| Time to fatigue during static handgrip at 40% of maximum (s) [16] |
| Counter movement jump height (cm) [25]  Maximal voluntary isometric strength of knee extensors and plantar flexors (Nm) with electrical stimulation [26]  Peak isokinetic torque of quadriceps and hamstrings at 30º.s (Nm) [27]  Force to flex the knee from 90° to 125° (N) [28] |
| Maximal voluntary isometric strength of the quadriceps and hamstrings (Nm.kg) [29]  Isometric hip strength (Nm) [30] |
| Work done during Mosso’s Ergograph test (J) [17] |
| Endurance time at 20, 40 and 60% handgrip maximum strength (s) [31] |
| Bench press mean weight lifted (pounds) [32]  Leg press mean weight lifted (pounds) [32]  MVC (kg) during leg press exercise [33]  Mean and peak force at 20, 40, 60 and 80% of one-repetition maximum performed on a Smith Machine [34]  Maximum voluntary isometric strength of the quadriceps (N) [9]  Jump height (cm) from a drop jump [35]  Maximum isometric torque of knee extensor muscles (Nm) with electrical stimulation [35]  Maximum voluntary contraction (Nm) [36]  Mean time to task failure (s) during an endurance task [36]  Absolute performance during a five-jump test (m) [37]  Standing broad jump performance distance (inches) [38]  Maximum hip flexion and extension strength (pounds) [38] |
| Endurance |
| Cycling TTE at 70% $\dot{\boldsymbol{V}}$O_2peak_ (min) [39]  Queens College Step Test to predict $\dot{\boldsymbol{V}}$O_2max_ (ml.kg^-^1.min^-1^) [40]  Treadmill running TTE at a heart rate of 135–140 b.min^-1^ (min) [40]  $\dot{\boldsymbol{V}}$O_2peak_ (ml.kg-1.min-1) during a progressive-intensity, continuous, treadmill running test to exhaustion [41]  Treadmill running TTE at 70% $\dot{\boldsymbol{V}}$O_2peak_ (min) [41]  $\dot{\boldsymbol{V}}$O_2max_ (ml.kg^-^1.min^-1^) during a progressive incremental exercise test on a treadmill until exhaustion [42] |
| TTE during a progressive incremental exercise test on a treadmill (min) [42] |
| v$\dot{\boldsymbol{V}}$O_2max_ (km.h) during an incremental maximal test to exhaustion performed on a treadmill [43]  Peak treadmill velocity (km.h) during an incremental maximal test to exhaustion [43] |
| Anaerobic capacity (W) from a Wingate Test [44, 45, 46]  Peak power (W) from a Wingate Test [44, 45, 46]  Power decline (W) from a Wingate Test [44]  Margaria-Kalamen (kgm.s) [44] |
| Cycled for 2 hours at 70% $\dot{\boldsymbol{V}}$O_2peak_ and then completed a 4 kJ/kg body weight TT on a cycle ergometer (min) [47] |
| Power output (W) during a continuously graded incremental test until exhaustion on a cycle ergometer [48]  TTE (min) during a continuously graded incremental test until exhaustion on a cycle ergometer [48]  $\dot{\boldsymbol{V}}$O_2peak_ (l.min) from a continuously graded incremental test until exhaustion on a cycle ergometer [48]  $\dot{\boldsymbol{V}}$O_2max_ (ml.kg-1.min-1) from an incremental graded-exercise test until volitional exhaustion on a cycle ergometer [49]  TTE (min) from an incremental graded-exercise test until volitional exhaustion on a cycle ergometer [49]  $\dot{\boldsymbol{V}}$O_2max_ (l.min^-1^) from a progressively increasing protocol for nine minutes on a cycle ergometer [50]  Working capacity at a heart rate of 170 b.min (W) from a progressively increasing protocol for nine minutes on a cycle ergometer [50]  Maximal pedalling time (s) from a progressively increasing protocol for nine minutes on a cycle ergometer [50]  $\dot{\boldsymbol{V}}$O_2max_ (ml.kg-1.min-1) from a maximal incremental exercise treadmill protocol until exhaustion [51]  TTE (min) from a maximal incremental exercise treadmill protocol until exhaustion [51]  $\dot{\boldsymbol{V}}$O_2max_ (ml.kg-1.min-1) from an incremental test on a cycle ergometer until exhaustion [52, 53, 54]  TTE (s) during a progressive maximal exercise test performed on a cycle ergometer [54]  $\dot{\boldsymbol{V}}$O_2max_ (ml.kg-1.min-1) [55]  TTE during a 1.5-mile run-walk (s) [55]  TTE during a 600-yard run-walk (s) [55]  Distance covered during a 12-minute run-walk (miles) [55]  $\dot{\boldsymbol{V}}$O_2max_ (ml.kg-1.min-1) during a graded exercise test on a cycle ergometer until exhaustion [56] |
| Maximal cycling power (W) during a force-velocity test [20]  Optimal velocity (rpm) during a force-velocity test [20]  Optimal force (kg) during a force-velocity test [20]  Physical working capacity (kg.m.min) [57]  $\dot{\boldsymbol{V}}$O_2max_ (l.min^-1^) from an incremental stress test until exhaustion on a cycle ergometer [58, 59, 60]  TTE (s) from an incremental stress test until exhaustion on a cycle ergometer [58, 59, 60]  Maximum power output (W) from an incremental stress test until exhaustion on a cycle ergometer [58, 59] |
| $\dot{\boldsymbol{V}}$O_2max_ (ml.kg-1.min-1) from a maximal progressive incremental test until exhaustion on a cycle ergometer [61, 62]  TTE (min) during a prolonged exercise performance test on a cycle ergometer at 60% $\dot{\boldsymbol{V}}$O_2max_ followed by an incremental exercise test until exhaustion [63] |
| Sprint time (s) at 5, 10 and 30 m [25]  Distance covered during Yo-Yo Intermittent Endurance Test (m) [25, 37] |
| Maximum power output (kpm.min) during a progressive incremental exercise test to exhaustion on a cycle ergometer [64]  Cycling TTE at 90% W_max_ (min) [64]  Peak power (W.kg), from a Wingate test [65]  Mean power (W.kg) from a Wingate test [65]  Fatigue index (%) from a Wingate test [45, 46, 65] |
| Peak cycling power (W.kg) during a ramp test on a cycle ergometer until exhaustion [66]  $\dot{\boldsymbol{V}}$O_2max_ (l.min^-1^) from a continuous progressive test until exhaustion on a treadmill [27]  Anaerobic speed test (s) [27]  TTE during an endurance run at 90% $\dot{\boldsymbol{V}}$O_2max_ (s) [27]  TTE (s) during a 20 s repeat sprint continuous incremental protocol until exhaustion on a treadmill [67]  TTE (min) during a continuous incremental exercise protocol on a treadmill [68]  16 km TT performance (min) on a cycle ergometer [69]  15 km TT performance (min) on a cycle ergometer [70]  30 km TT performance (min) on a cycle ergometer [70] |
| Tennis serve performance accuracy [30]  Tennis serve performance velocity (mph) [30] |
| 100-m freestyle time (s) [32]  200-m freestyle time (s) [32]  Peak power output (W) from an incremental exercise test on a cycle ergometer [60]  Total work done (kJ) from an incremental exercise test on a cycle ergometer [60] |
| Sprint duration until exhaustion throughout maximum accumulated oxygen deficit tests on a cycle ergometer [71]  Power relative (W.kg) from an incremental test until voluntary exhaustion on a cycle ergometer [72]  $\dot{\boldsymbol{V}}$O_2max_ (ml.min) from an incremental test until voluntary exhaustion on a cycle ergometer [72]  Distance ran during Loughborough Intermittent Shuttle Test (m) [73]  15 m sprint time (s) [73] |
| Mean and peak power outputs during an all-out 30 second sprint (W) [74] |
| TTE (min) from an incremental maximal exercise test on a cycle ergometer [75]  Repeated shuttle-sprint ability test mean time (s) [37] |
| Peak and mean power output during repeat sprint tests (W) [76]  Fatigue index for power during repeat sprint tests (%) [76]  Peak and mean speed during repeat sprint tests (m.s) [76]  Fatigue index for speed during repeat sprint tests (m.s) [76] |
| Incremental rowing ergometer test to determine $\dot{\boldsymbol{V}}$O_2max_ (l.min) [77]  Incremental rowing ergometer test to determine maximal power output (W) [77] |
| Time of attaining anaerobic peak power during maximal cycling sprint test (s) [78]  Time of maintaining anaerobic peak power during maximal cycling sprint test (s) [78]  Power decrease during maximal cycling sprint test (W.kg.s) [78]  Peak cycling power during an incremental test on a cycle ergometer until exhaustion (W.kg) [78] |

TTE, time to exhaustion; MILS, maximal isometric lifting strength; $\dot{\boldsymbol{V}}$O_2max_ maximal oxygen uptake; $\dot{\boldsymbol{V}}$O_2peak_ peak oxygen uptake; v$\dot{\boldsymbol{V}}$O_2max,_ velocity at maximal oxygen uptake.

Please note that exact duplicate outcomes were deleted.

*References*

1. Abt JP, Sell TC, Laudner KG, McCrory JL, Loucks TL, Berga SL, et al. Neuromuscular and biomechanical characteristics do not vary across the menstrual cycle. Knee Surg Sports Traumatolo Arthrosc. 2007;15(7):901-7. <https://doi.org/10.1007/s00167-007-0302-3>
2. Ansdell P, Brownstein CG, Škarabot J, Hicks KM, Simoes DC, Thomas K, et al. Menstrual cycle-associated modulations in neuromuscular function and fatigability of the knee extensors in eumenorrheic women. J Appl Physiol. 2019; 126(6):1701-12. <https://doi.org/10.1152/japplphysiol.01041.2018>
3. Bambaeichi E, Reilly T, Cable NT, Giacomoni M. The isolated and combined effects of menstrual cycle phase and time-of-day on muscle strength of eumenorrheic females. Chronobiol Int. 2004;21(4-5):645-60. <https://doi.org/10.1081/CBI-120039206>
4. Bell DR, Blackburn JT, Ondrak KS, Hackney AC, Hudson JD, Norcross MF, et al. The effects of oral contraceptive use on muscle stiffness across the menstrual cycle. Clin J Sport Med. 2011;21(6):467-73. <https://doi.org/10.1097/JSM.0b013e318230f50a>
5. ^a^Birch KM, Reilly T. Manual handling performance: the effects of menstrual cycle phase. Ergonomics. 1999;42(10):1317-32.
6. ^b^Birch K, Reilly T. The diurnal rhythm in isometric muscular performance differs with eumenorrheic menstrual cycle phase. Chronobiol Int. 2002;19(4):731-42. <https://doi.org/10.1081/CBI-120006083>
7. Davies BN, Elford JC, Jamieson KF. Variations in performance in simple muscle tests at different phases of the menstrual cycle. J Sports Med Phys Fitness. 1991;31(4):532-7.
8. ^a^Janse de Jonge X, Boot CR, Thom JM, Ruell PA, Thompson MW. The influence of menstrual cycle phase on skeletal muscle contractile characteristics in humans. J Physiol. 2001;530(1):161-6.<https://doi.org/10.1111/j.1469-7793.2001.0161m.x>
9. Sarwar R, Niclos BB, Rutherford OM. Changes in muscle strength, relaxation rate and fatiguability during the human menstrual cycle. J Physiol. 1996;493(1):267-72.
10. Dibrezzo RO, Fort IL, Brown B. Dynamic strength and work variations during three stages of the menstrual cycle. J Orthop Sports Phys Ther. 1988;10(4):113-6.
11. Drake SM, Evetovich T, Eschbach C, Webster M. A pilot study on the effect of oral contraceptives on electromyography and mechanomyography during isometric muscle actions. J Electromyogr Kinesiol. 2003;13(3):297-301. <https://doi.org/10.1016/S1050-6411(03)00024-5>
12. Ekenros L, Hirschberg AL, Heijne A, Fridén C. Oral contraceptives do not affect muscle strength and hop performance in active women. Clin J Sport Med. 2013;23(3):202-7.<https://doi.org/10.1097/JSM.0b013e3182625a51>
13. Fridén C, Hirschberg AL, Saartok T. Muscle strength and endurance do not significantly vary across 3 phases of the menstrual cycle in moderately active premenopausal women. Clin J Sport Med. 2003;13(4):238-41.
14. Ettinger SM, Silber DH, Gray KS, Smith MB, Yang QX, Kunselman AR, et al. Effects of the ovarian cycle on sympathetic neural outflow during static exercise. J Appl Physiol. 1998;85(6):2075-81.
15. Hoshi A. Changes in muscular strength of women in different phases of the menstrual cycle. Bulletin of the Nippon Dental University. 1997;26:219-24.
16. Jarvis SS, VanGundy TB, Galbreath MM, Shibata S, Okazaki K, Reelick MF, et al. Sex differences in the modulation of vasomotor sympathetic outflow during static handgrip exercise in healthy young humans. Am J Physiol Regul Integr Comp Physiol. 2011;301(1):193-200.
17. Pallavi LC, SoUza UJ, Shivaprakash G. Assessment of musculoskeletal strength and levels of fatigue during different phases of menstrual cycle in young adults. J Clin Diagn Res. 2017;11(2):11-13.
18. ^a^Elliott KJ, Cable NT, Reilly T, Diver MJ. Effect of menstrual cycle phase on the concentration of bioavailable 17-β oestradiol and testosterone and muscle strength. Clin Sci. 2003;105(6):663-9. <https://doi.org/10.1042/CS20020360>
19. ^b^Elliott KJ, Cable NT, Reilly T. Does oral contraceptive use affect maximum force production in women?. Br J Sports Med. 2005;39(1):15-9. <http://dx.doi.org/10.1136/bjsm.2003.009886>
20. Giacomoni M, Bernard T, Gavarry O, Altare S, Falgairette G. Influence of the menstrual cycle phase and menstrual symptoms on maximal anaerobic performance. Med Sci Sports Exerc. 2000;32(2):486.
21. ^b^Gordon D, Scruton A, Barnes R, Baker J, Prado L, Merzbach V. The effects of menstrual cycle phase on the incidence of plateau at and associated cardiorespiratory dynamics. Clin Physiol Funct Imaging. 2018; 38(4):689-98. <https://doi.org/10.1111/cpf.12469>
22. Gür H. Concentric and eccentric isokinetic measurements in knee muscles during the menstrual cycle: a special reference to reciprocal moment ratios. Arch Phys Med Rehabil. 1997;78(5):501-5.
23. Hertel J, Williams NI, Olmsted-Kramer LC, Leidy HJ, Putukian M. Neuromuscular performance and knee laxity do not change across the menstrual cycle in female athletes. Knee Surg Sports Traumatol Arthrosc. 2006;14(9):817-22. <https://doi.org/10.1007/s00167-006-0047-4>
24. Hoeger Bement MK, Rasiarmos RL, DiCapo JM, Lewis A, Keller ML, Harkins AL, et al. The role of the menstrual cycle phase in pain perception before and after an isometric fatiguing contraction. Eur J Appl Physiol. 2009;106(1):105-12.
25. Julian R, Hecksteden A, Fullagar HH, Meyer T. The effects of menstrual cycle phase on physical performance in female soccer players. PloS one. 2017;12(3):e0173951. <https://doi.org/10.1371/journal.pone.0173951>
26. Kubo K, Miyamoto M, Tanaka S, Maki A, Tsunoda N, Kanehisa H. Muscle and tendon properties during menstrual cycle. Int J Sports Med. 2009;30(2):139-43. <https://doi.org/10.1055/s-0028-1104573>
27. Lebrun CM, McKenzie DC, Prior JC, Taunton JE. Effects of menstrual cycle phase on athletic performance. Med Sci Sports Exerc. 1995;27(3):437-44.
28. Lee H, Petrofsky JS, Daher N, Berk L, Laymon M. Differences in anterior cruciate ligament elasticity and force for knee flexion in women: oral contraceptive users versus non-oral contraceptive users. Euro J Appl Physiol. 2014;114(2):285-94. <https://doi.org/10.1007/s00421-013-2771-z>
29. Montgomery MM, Shultz SJ. Isometric knee-extension and knee-flexion torque production during early follicular and postovulatory phases in recreationally active women. J Athl Train. 2010;45(6):586-93.
30. Otaka M, Chen SM, Zhu Y, Tsai YS, Tseng CY, Fogt DL, et al. Does ovulation affect performance in tennis players?. Br J Sports Med. 2018;4(1):e000305. <http://dx.doi.org/10.1136/bmjsem-2017-000305>
31. Petrofsky J, Al Malty A, Suh HJ. Isometric endurance, body and skin temperature and limb and skin blood flow during the menstrual cycle. Med Sci Moni. 2007;13(3):111-7.
32. Quadagno D, Faquin L, Lim GN, Kuminka W, Moffatt R. The menstrual cycle: does it affect athletic performance?. Physician Sports Med. 1991;19(3):121-4.
33. Rodrigues P, de Azevedo Correia M, Wharton L. Effect of menstrual cycle on muscle strength. J Exerc Physiol Online. 2019;22(5):89-96.
34. Romero-Moraleda B, Del Coso J, Gutiérrez-Hellín J, Ruiz-Moreno C, Grgic J, Lara B. The influence of the menstrual cycle on muscle strength and power performance. J Hum Kinet. 2019;68:123-133.
35. Sipavičienė S, Daniusevičiutė L, Klizienė I, Kamandulis S, Skurvydas A. Effects of estrogen fluctuation during the menstrual cycle on the response to stretch-shortening exercise in females. BioMed Res Int. 2013; 2013:1-6. <http://dx.doi.org/10.1155/2013/243572>
36. Tenan MS, Hackney AC, Griffin L. Maximal force and tremor changes across the menstrual cycle. Eur J Appl Physiol. 2016;116(1):153-60. <https://doi.org/10.1007/s00421-015-3258-x>
37. Tounsi M, Jaafar H, Aloui A, Souissi N. Soccer-related performance in eumenorrheic Tunisian high-level soccer players: effects of menstrual cycle phase and moment of day. J Sports Med Phys Fitness. 2018;58(4):497-502. <https://doi.org/10.23736/s0022-4707.17.06958-4>
38. Wearing MP, Yuhosz MD, Campbell R, Love EJ. The effect of the menstrual cycle on tests of physical fitness. J Sports Med Phys Fitness. 1972;12(1):38-41.
39. Bailey SP, Zacher CM, Mittleman KD. Effect of menstrual cycle phase on carbohydrate supplementation during prolonged exercise to fatigue. J Appl Physiol. 2000;88(2):690-7. <https://doi.org/10.1152/jappl.2000.88.2.690>
40. Bandyopadhyay A, Dalui R. Endurance capacity and cardiorespiratory responses in sedentary females during different phases of menstrual cycle. **Kathmandu** Univ Med J. 2012;10(4):25-9. [https://doi.org/10.3126/kumj.v10i4.10990](https://doi.org/10.3126/kumj.v10i4.10990%20%20)
41. Beidleman BA, Rock PB, Muza SR, Fulco CS, Forte Jr VA, Cymerman A. Exercise V̇e and physical performance at altitude are not affected by menstrual cycle phase. J Appl Physiol. 1999;86(5):1519-26. <https://doi.org/10.1152/jappl.1999.86.5.1519>
42. Bemben DA, Salm PC, Salm AJ. Ventilatory and blood lactate responses to maximal treadmill exercise during the menstrual cycle. J Sports Med Phys Fitness. 1995;35(4):257-62.
43. Burrows M, Bird SR. Velocity at $\dot{V}$O_2max_ and peak treadmill velocity are not influenced within or across the phases of the menstrual cycle. Euro J Appl Physiol. 2005;93(5-6):575-80. <https://doi.org/10.1007/s00421-004-1272-5>
44. Bushman B, Masterson G, Nelsen J. Anaerobic power performance and the menstrual cycle: eumenorrheic and oral contraceptive users. J Sports Med Phys Fitness. 2006;46(1):132.
45. Masterson G. The impact of menstrual phases on anaerobic power performance in collegiate women. J Strength Cond Res. 1999;13(4):325-9.
46. Okudan N, Gokbel H, Ucok K, Baltaci A. Serum leptin concentration and anaerobic performance do not change during the menstrual cycle of young females. Neuroendocrinology Letters. 2005;26(4):297-300.
47. Campbell SE, Angus DJ, Febbraio MA. Glucose kinetics and exercise performance during phases of the menstrual cycle: effect of glucose ingestion. Am J Physiol Endocrinol Metab. 2001;281(4):817-25. <https://doi.org/10.1152/ajpendo.2001.281.4.E817>
48. Casazza GA, Suh SH, Miller BF, Navazio FM, Brooks GA. Effects of oral contraceptives on peak exercise capacity. J Appl Physiol. 2002;93(5):1698-702. <https://doi.org/10.1152/japplphysiol.00622.2002>
49. Dean TM, Perreault L, Mazzeo RS, Horton TJ. No effect of menstrual cycle phase on lactate threshold. J Appl Physiol. 2003;95(6):2537-43. <https://doi.org/10.1152/japplphysiol.00672.2003>
50. de Bruyn-Prevost P, Masset C, Sturbois X. Physiological response from 18-25 years women to aerobic and anaerobic physical fitness tests at different periods during the menstrual cycle. J Sports Med. 1984;24(2):144-8.
51. de Souza MS, Maguire MS, Rubin KR, Maresh CM. Effects of menstrual phase and amenorrhea on exercise performance in runners. Med Sci Sports Exerc. 1990;22(5):575-80. <https://doi.org/10.1249/00005768-199010000-00006>
52. Dombovy ML, Bonekat HW, Williams TJ, Staats BA. Exercise performance and ventilatory response in the menstrual cycle. Med Sci Sports Exerc. 1987;19(2):111-7.
53. Kaygisiz Z, Erkasap N, Soydan M. Cardiorespiratory responses to submaximal incremental exercise are not affected by one night's sleep deprivation during the follicular and luteal phases of the menstrual cycle. Indian J Pharmacol. 2003;47(3):279-87.
54. Kraemer WJ, Kim SK, Bush JA, Nindl BC, Volek JS, Spiering BA, et al. Influence of the menstrual cycle on proenkephalin peptide F responses to maximal cycle exercise. Euro J Appl Physiol. 2006;96(5):581-6.
55. Doolittle TL, Engebretsen J. Performance variations during the menstrual cycle. J Sports Med Phys Fitness. 1972;12(1):54.
56. Frandsen J, Pistoljevic N, Quesada JP, Amaro-Gahete FJ, Ritz C, Larsen S, et al. Menstrual cycle phase does not affect whole body peak fat oxidation rate during a graded exercise test. J Appl Physiol. 2020;128(3):681-7. <https://doi.org/10.1152/japplphysiol.00774.2019>
57. Girija B, Veeraiah SH. Effect of different phases of menstrual cycle on physical working capacity in Indian population. Indian J Physiol Pharmacol. 2011;55(2):165-9.
58. ^b^Gordon D, Scruton A, Barnes R, Baker J, Prado L, Merzbach V. The effects of menstrual cycle phase on the incidence of plateau at and associated cardiorespiratory dynamics. Clin Physiol Funct Imaging. 2018; 38(4):689-98. <https://doi.org/10.1111/cpf.12469>
59. Mattu AT, Iannetta D, MacInnis MJ, Doyle‐Baker PK, Murias JM. Menstrual and oral contraceptive cycle phases do not affect submaximal and maximal exercise responses. Scand J Med Sci Sports. 2019. <https://doi.org/10.1111/sms.13590>
60. Redman LM, Scroop GC, Norman RJ. Impact of menstrual cycle phase on the exercise status of young, sedentary women. Euro J Appl Physiol. 2003;90(5-6):505-13. <https://doi.org/10.1007/s00421-003-0889-0>
61. ^a^Grucza R, Pekkarinen H, Titov EK, Kononoff A, Hänninen O. Influence of the menstrual cycle and oral contraceptives on thermoregulatory responses to exercise in young women. Eur J Appl Physiol Occup Physiol. 1993;67(3):279-85.
62. ^b^Grucza R, Pekkarinen H, Hanninen O. Cardiorespiratory responses to bicycle incremental exercise in women taking oral contraceptives. Biol Sport. 2002;19(3):267-79.
63. ^b^Janse de Jonge X, Thompson MW, Chuter VH, Silk LN, Thom JM. Exercise performance over the menstrual cycle in temperate and hot, humid conditions. Med Sci Sports Exerc. 2012;44(11):2190-8. <https://doi.org/10.1249/mss.0b013e3182656f13>
64. Jurkowski JE, Jones NL, Toews CJ, Sutton JR. Effects of menstrual cycle on blood lactate, O2 delivery, and performance during exercise. J Appl Physiol. 1981;51(6):1493-9.
65. ^a^Lara B, Gutiérrez Hellín J, Ruíz‐Moreno C, Romero‐Moraleda B, Del Coso J. Acute caffeine intake increases performance in the 15‐s Wingate test during the menstrual cycle. Br J Clin. 2019;86:745-752. <https://doi.org/10.1111/bcp.14175>
66. ^b^Lara B, Gutiérrez-Hellín J, García-Bataller A, Rodríguez-Fernández P, Romero-Moraleda B, Del Coso J. Ergogenic effects of caffeine on peak aerobic cycling power during the menstrual cycle. Euro J Nutr. 2019;1-10. <https://doi.org/10.1007/s00394-019-02100-7>
67. Lynch NJ, Nimmo MA. Effects of menstrual cycle phase and oral contraceptive use on intermittent exercise. Eur J Appl Physiol Occup Physiol. 1998;78(6):565-72.
68. McCracken M, Ainsworth B, Hackney AC. Effects of the menstrual cycle phase on the blood lactate responses to exercise. Eur J Appl Physiol Occup Physiol. 1994;69(2):174-5.
69. McLay RT, Thomson CD, Williams SM, Rehrer NJ. Carbohydrate loading and female endurance athletes: effect of menstrual-cycle phase. Int J Sport Nutr Exerc Metab. 2007;17(2):189-205. <https://doi.org/10.1123/ijsnem.17.2.189>
70. Oosthuyse T, Bosch AN, Jackson S. Cycling time trial performance during different phases of the menstrual cycle. Euro J Appl Physiol. 2005;94(3):268-76. 10.1007/s00421-005-1324-5
71. Shaharudin S, Ghosh AK, Ismail AA. Anaerobic capacity of physically active eumenorrheic females at mid-luteal and mid-follicular phases of ovarian cycle. J Sports Med Phys Fitness. 2011;51(4):576.
72. Smekal G, Von Duvillard SP, Frigo P, Tegelhofer T, Pokan R, Hofmann P, et al. Menstrual cycle: no effect on exercise cardiorespiratory variables or blood lactate concentration. Med Sci Sports Exerc. 2007;39(7):1098-106. <https://doi.org/10.1249/mss.0b013e31805371e7>
73. ^a^Sunderland C, Nevill M. Effect of the menstrual cycle on performance of intermittent, high-intensity shuttle running in a hot environment. Euro J Appl Physiol. 2003;88(4-5):345-52. <https://doi.org/10.1007/s00421-002-0722-1>
74. ^b^Sunderland C, Tunaley V, Horner F, Harmer D, Stokes KA. Menstrual cycle and oral contraceptives’ effects on growth hormone response to sprinting. Appl Physiol Nutr Metab. 2011;36(4):495-502. <https://doi.org/10.1139/h11-039>
75. Takase K, Nishiyasu T, Asano K. Modulating effects of the menstrual cycle on cardiorespiratory responses to exercise under acute hypobaric hypoxia. Japanese J Physiol. 2002;52(6):553-60. <https://doi.org/10.2170/jjphysiol.52.553>
76. Tsampoukos A, Peckham EA, James R, Nevill ME. Effect of menstrual cycle phase on sprinting performance. Eur J Appl Physiol. 2010;109(4):659-67. <https://doi.org/10.1007/s00421-010-1384-z>
77. Vaiksaar S, Jürimäe J, Mäestu J, Purge P, Kalytka S, Shakhlina L, et al. No effect of menstrual cycle phase and oral contraceptive use on endurance performance in rowers. J Strength Cond Res. 2011;25(6):1571-8. <https://doi.org/10.1519/JSC.0b013e3181df7fd2>
78. Wiecek M, Szymura J, Maciejczyk M, Cempla J, Szygula Z. Effect of sex and menstrual cycle in women on starting speed, anaerobic endurance and muscle power. Acta Physiologica Hungarica. 2016;103(1):127-32. <https://doi.org/10.1556/036.103.2016.1.13>
