# Supplementary material for: The Effects of Menstrual Cycle Phase on Exercise Performance in Eumenorrheic Women: A Systematic Review and Meta-Analysis
Source: Sports Med. 2020 Jul 13;50(10):1813–27. doi: 10.1007/s40279-020-01319-3 (PMC7497427; doi:10.1007/s40279-020-01319-3)
Supplement: Supplementary file 3 — Supplementary material 3 (DOCX 14 kb) [file 40279_2020_1319_MOESM3_ESM.docx]

The Effects of Menstrual Cycle Phase on Exercise Performance in Eumenorrheic Women: A Systematic Review and Meta-Analysis. Sports Medicine. Corresponding author: Dr Kirsty Elliott-Sale, Sport Health and Performance Enhancement (SHAPE) Research Centre, Department of Sport Science, Nottingham Trent University, Nottingham, UK. Email: kirsty.elliottsale@ntu.ac.uk.

**Electronic Supplementary Material Appendix S3.** Example of a Search Strategy Conducted in PubMed (14/01/2019).

| **Limits applied** | |  |
| --- | --- | --- |
| Humans | |  |
| Females | |  |
| English language | |  |
| **Search terms** | **Number of results** | |
| Menstrual cycle OR menstrual phase OR follicular phase OR luteal phase and athletic performance | 221 | |
| Menstrual cycle OR menstrual phase OR follicular phase OR luteal phase and sports performance | 277 | |
| Menstrual cycle OR menstrual phase OR follicular phase OR luteal phase and strength | 197 | |
| Menstrual cycle OR menstrual phase OR follicular phase OR luteal phase and torque | 16 | |
| Menstrual cycle OR menstrual phase OR follicular phase OR luteal phase and force | 144 | |
| Menstrual cycle OR menstrual phase OR follicular phase OR luteal phase and max* voluntary contraction | 24 | |
| Menstrual cycle OR menstrual phase OR follicular phase OR luteal phase and isometric | 55 | |
| Menstrual cycle OR menstrual phase OR follicular phase OR luteal phase and isokinetic | 16 | |
| Menstrual cycle OR menstrual phase OR follicular phase OR luteal phase and neuromuscular | 46 | |
| Menstrual cycle OR menstrual phase OR follicular phase OR luteal phase and skeletal muscle | 184 | |
| Menstrual cycle OR menstrual phase OR follicular phase OR luteal phase and muscular performance | 14 | |
| Menstrual cycle OR menstrual phase OR follicular phase OR luteal phase and power | 285 | |
| Menstrual cycle OR menstrual phase OR follicular phase OR luteal phase and anaerobic | 65 | |
| Menstrual cycle OR menstrual phase OR follicular phase OR luteal phase and anaerobic capacity | 9 | |
| Menstrual cycle OR menstrual phase OR follicular phase OR luteal phase and anaerobic power | 12 | |
| Menstrual cycle OR menstrual phase OR follicular phase OR luteal phase and aerobic | 119 | |
| Menstrual cycle OR menstrual phase OR follicular phase OR luteal phase and endurance | 158 | |
| Menstrual cycle OR menstrual phase OR follicular phase OR luteal phase and aerobic capacity | 27 | |
| Menstrual cycle OR menstrual phase OR follicular phase OR luteal phase and aerobic power | 19 | |
| Menstrual cycle OR menstrual phase OR follicular phase OR luteal phase and endurance capacity | 26 | |
| Menstrual cycle OR menstrual phase OR follicular phase OR luteal phase and endurance power | 13 | |
| **Total: 1927 (with duplicates)** | | |
