# Supplementary material for: The Effects of Menstrual Cycle Phase on Exercise Performance in Eumenorrheic Women: A Systematic Review and Meta-Analysis
Source: Sports Med. 2020 Jul 13;50(10):1813–27. doi: 10.1007/s40279-020-01319-3 (PMC7497427; doi:10.1007/s40279-020-01319-3)
Supplement: Supplementary file 4 — Supplementary material 4 (DOCX 16 kb) [file 40279_2020_1319_MOESM4_ESM.docx]

The Effects of Menstrual Cycle Phase on Exercise Performance in Eumenorrheic Women: A Systematic Review and Meta-Analysis. Sports Medicine. Corresponding author: Dr Kirsty Elliott-Sale, Sport Health and Performance Enhancement (SHAPE) Research Centre, Department of Sport Science, Nottingham Trent University, Nottingham, UK. Email: kirsty.elliottsale@ntu.ac.uk.

**Electronic Supplementary Material Appendix S4.** Study Quality Assessment.

| **Downs and Black checklist (maximum score attainable = 16). Study quality was categorised as follows: “high”: 14 – 16; “moderate”: 10 – 13; “low”: 6 – 9; “very low”: 0 - 5).** | |
| --- | --- |
| *Reporting* | |
| Q1. | Is the hypothesis/aim/objective of the study clearly described?  Yes = 1  No = 0 |
| Q2. | Are the main outcomes to be measured clearly described in the introduction or methods section? If the main outcomes are first mentioned in the results section, answer no.  Yes = 1  No = 0 |
| Q3. | Are the characteristics of the participants included in the study clearly described? In observational studies, inclusion and/or exclusion criteria should be given. In case-control studies, inclusion and/or exclusion and the source of controls should be given.  Yes = 1  No = 0 |
| Q4. | Were the tested menstrual cycle phases clearly described? Answer yes if the precise criteria used to define phase were provided, answer no if the exact phase tested cannot be ascertained (*e.g.,* vague language such as “early” or “late” were used, without defining the criteria)  Yes = 1  No = 0 |
| Q5. | Are the main findings of the study clearly described? Simple outcome data should be reported for all major findings so the reader can check the major analyses and conclusions. This does not cover statistical tests which are addressed in other questions.  Yes = 1  No = 0 |
| Q6. | Does the study provide estimates of the random variability in the data for the main outcomes? In non-normal data, inter-quartile range should be reported. In normal data, standard deviation, standard error or confidence intervals should be reported.  Yes = 1  No = 0 |
| *External validity* | |
| Q7. | Were the subjects confirmed as non-hormonal contraceptive users, for at least three months prior to participation?  Yes = 1  No = 0  Unable to determine = 0 |
| *Internal validity – bias* | |
| Q8. | Was at least one familiarization trial conducted prior to exercise testing?  Yes = 1  No = 0  Unable to determine = 0 |
| Q9. | Were the exercise test conditions adequately standardised (taking into consideration factors including time of day, prior nutritional intake [including caffeine] and prior exercise).  Yes (all relevant factors standardised) = 2  Yes (some relevant factors standardised) = 1  Exercise testing unstandardized = 0  Unable to determine = 0 |
| Q.10 | If any of the results of the study were based on ‘data dredging’ was this made clear? Any analyses that had not been planned at the outset should be clearly indicated. If no retrospective subgroup analyses were reported, then answer yes.  Yes = 1  No = 0  Unable to determine = 0 |
| Q11. | Were statistical tests used to assess the main outcomes appropriate? The statistical techniques used must be appropriate to the data and the research question.  Yes = 1  No = 0  Unable to determine = 0 |
| Q12. | Were the main outcome measures used accurate (*i.e.,* valid and reproducible)? For studies where the validity and reproducibility of outcome measures are clearly described, the question should be answered yes. For studies which refer to other work that demonstrates the outcome measures are accurate, answer yes.  Yes = 1  No = 0  Unable to determine = 0 |
| *Internal validity – confounding (selection bias)* | |
| Q.13 | Was the order of phase testing randomised?  Yes = 1  No = 0  Unable to determine = 0 |
| *Power* | |
| Q.14 | Did the study have sufficient power to detect an *a priori* specified scientifically important effect at a pre-determined probability threshold? Answer yes if they included a power calculation, and no if not.  Yes = 1  No = 0 |
| Q15. | Was study retention > 85%?  Yes = 1  No = 0  Unable to determine = 0 |
| **GRADE (assign an *a-priori* study quality rating based on the modified Downs and Black checklist, so all studies will start out as being of “high”, “moderate”, “low”, “very low”).** | |
| Q1. | Identify if menstrual cycle phase was confirmed using blood samples. If yes, the *a priori* rating is maintained and this is the final study quality rating. If not, the study is downgraded a level (*e.g.,* a study that started out as high, drops to moderate). |
| Q2. | Identify if menstrual cycle phase was confirmed using ovulation kits. If yes, the Q1. rating is maintained. If no the study is downgraded another level (*e.g.,* a study that started out high, drops to low). This means that the maximum rating that any study that does not use blood analysis or ovulation kits is “low” or “very low”. |
