# Supplementary material for: The Effects of Menstrual Cycle Phase on Exercise Performance in Eumenorrheic Women: A Systematic Review and Meta-Analysis
Source: Sports Med. 2020 Jul 13;50(10):1813–27. doi: 10.1007/s40279-020-01319-3 (PMC7497427; doi:10.1007/s40279-020-01319-3)
Supplement: Supplementary file 5 — Supplementary material 5 (DOCX 64 kb) [file 40279_2020_1319_MOESM5_ESM.docx]

The Effects of Menstrual Cycle Phase on Exercise Performance in Eumenorrheic Women: A Systematic Review and Meta-Analysis. Sports Medicine. Corresponding author: Dr Kirsty Elliott-Sale, Sport Health and Performance Enhancement (SHAPE) Research Centre, Department of Sport Science, Nottingham Trent University, Nottingham, UK. Email: kirsty.elliottsale@ntu.ac.uk.

**Electronic Supplementary Material Appendix S5.** Table of Included Studies.

| Author and date | Aim | Population (participant health, training status and sample size) | MC phases tested | Methods of determining MC phase | Outcome measure(s) | Study conclusion | Quality rating |
| --- | --- | --- | --- | --- | --- | --- | --- |
| Abt *et al.* (2007) [1] | To determine whether MC phase affects fine motor coordination, postural stability, knee strength and knee joint kinematics and kinetics | Physically active females (n = 10) | EF, ovulation and ML | Counting of days, MC history, urinary ovulation detection test and serum oestrogen and progesterone | Hamstring:quadricep strength ratio at 60 and 180 s^–1^ | No differences existed between phases of the MC for hamstring:quadriceps strength ratio at 60 and 180 s^–1^ | Moderate |
| Ansdell *et al.* (2019) [2] | To investigate knee extensor neuromuscular function and fatigability across the MC | Eumenorrheic females (n = 13) | EF, ovulation and ML | Counting of days, MC history, and serum oestrogen and progesterone | MVC with motor nerve stimulation (N)  Time to task failure during fatiguing task involving sets of intermittent isometric contractions (s) performed in the lower-body | MVC was not affected by MC phase. Time to task failure was longer in the ML phase compared to both the EF and ovulatory phases | Moderate |
| Bailey *et al.* (2000) [3] | To determine whether MC phase influences the effect of carbohydrate supplementation on substrate metabolism and fatigue during prolonged exercise | Moderately trained female cyclists (n = 9) | EF and ML | Counting of days and serum oestrogen and progesterone | Cycling TTE at 70% $\dot{V}$O_2peak_ (min) | No differences in TTE were observed between MC phases | Low |
| Bambaeichi *et al.* (2004) [4] | To determine whether the isolated and combined effects of circamensal variation and diurnal changes affect muscle strength | Sedentary females (n = 8) | EF, LF, ovulation, ML and LL | Counting of days, MC history, BBT (one month prior) and urinary ovulation detection test | Peak isokinetic torques of the knee flexors and extensors at 1.05 and 3.14 rad.s^-1^ (Nm) through 90º range of motion  Maximal voluntary isometric contraction of the knee flexors and extensors measured at (Nm) 0 rad.s^-1^ and 60º of knee flexion with and without electrical stimulation | MC phase variation was observed for peak torque of knee flexors at 1.05 and 3.14 rad.s (Nm) and also isometric contraction of knee flexors, with values being greatest at the ovulation phase | Low |
| Bandyopadhyay and Dalui (2012) [5] | To determine whether MC phase influences endurance capacity and cardiorespiratory responses | Sedentary females (n = 45) | EF, LF and ML | Counting of days and BBT | Queens College Step Test to predict $\dot{V}$O_2max_ (ml.kg^-1^.min^-1^)  Running TTE at a heart rate of 135–140 b.min^-1^ (min) | $\dot{V}$O_2max_ and running TTE were lower in the EF phase | Very low |
| Beidleman *et al.* (1999) [6] | To determine whether MC phase affects maximal and submaximal exercise performance at sea level and acute altitude | Physically active females (n = 8) | EF and ML | MC history, counting of days, urinary ovulation detection test and serum oestrogen and progesterone | $\dot{V}$O_2max_ (ml.kg^-1^.min^-1^ or l.min^-1^) during a progressive-intensity, continuous, treadmill running test to exhaustion  Running TTE at 70% $\dot{V}$O_2peak_ (min) | Neither $\dot{V}$O_2max_ nor running TTE was affected by MC phase | High |
| Bell *et al.* (2011) [7] | To determine whether MC phase affects hamstring neuro-mechanics and leg stiffness | Physically active females (n = 15) | EF and ovulation | Counting of days, MC history, urinary ovulation detection test and serum oestrogen and progesterone | Rate of force production during a maximal voluntary isometric hamstring contraction (N.s^-1^)  Time to 50% peak force during a maximal voluntary isometric hamstring contraction (ms) | No changes were observed across the MC for both variables | Moderate |
| Bemben *et al.* (1995) [8] | To determine whether MC phase affects ventilatory and blood lactate responses to maximal treadmill exercise | Moderately active women (n = 5) | EF, ovulation and ML | Coutning of days, BBT and serum oestrogen and progesterone | $\dot{V}$O_2max_ (ml.kg^-1^.min^-1^) and running TTE during a progressive incremental exercise test (min) | There were no differences in $\dot{V}$O_2max_ and running TTE between MC phases | Low |
| ^a^Birch and Reilly (1999) [9] | To determine whether MC phase affects the physical, physiological and subjective responses to both isometric and dynamic lifting performance | Healthy females (n = 17) | EF, LF, ovulation, ML and LL | Counting of days, MC history, BBT (two months prior and during), and assessment of symptoms and alterations in cervical mucus | MILS performed at both knee and waist height (N)  Time to volitional fatigue at 45% MILS performed at both knee and waist height (s)  Maximal acceptable load (kg) | No differences  between MC phases were identified for any of the lifting performances variables | Very low |
| ^b^Birch and Reilly (2002) [10] | To determine whether the circamensal and diurnal rhythms in temperature affect the production of maximal voluntary muscle force | Moderately physically active females (n = 10) | LF and ML | Counting of days, MC history, BBT and assessment of symptoms and alterations in cervical mucus | MILS at knee height (N)  Time to volitional fatigue at 45% MILS at knee height (min) | MILS and time to fatigue did not differ between either MC phase | Very low |
| Burrows and Bird (2005) [11] | To determine whether MC phase affects v$\dot{V}$O_2max_ and peak treadmill velocity in a homogenous group of highly trained female endurance runners | Highly trained endurance females (n = 10) | EF, LF, EL and LL | MC history, counting of days and salivary progesterone | v$\dot{V}$O_2max_ (km.h) and peak treadmill velocity (km.h) during an incremental maximal test to exhaustion performed on a treadmill | No differences in v$\dot{V}$O_2max_ or peak treadmill velocity were found between the phases of the MC | Low |
| Bushman *et al.* (2006) [12] | To determine whether MC phase affects short term, high intensity (power) performance in moderately active women | Active females (n = 7) | EF and EL | MC history, counting of days, BBT and urinary ovulation detection test | Anaerobic capacity, peak power and power decline from a Wingate Test (W)  Margaria-Kalamen (kgm.s) | There were no differences in Wingate or Margaria-Kalamen performance between MC phases | Low |
| Campbell *et al.* (2001) [13] | To determine whether MC phase and carbohydrate ingestion affects glucose kinetics and exercise performance | Healthy, moderately endurance-trained women (n = 8) | EF and ML | MC history, urinary ovulation detection test and serum oestrogen and progesterone | Cycled for 2 hrs at 70% $\dot{V}$O_2max_ and then completed a 4 kJ/kg body weight TT performance on a cycle ergometer (min) | TT performance was longer in the EF phase, compared to the ML phase of the MC | Moderate |
| Casazza *et al.* (2002) [14] | To determine whether MC phase affects peak exercise capacity, as measured by $\dot{V}$O_2peak_ | Healthy, habitually exercised females (n = 6) | LF and ML | MC history, counting of days, urinary ovulation detection test and serum oestrogen and progesterone | Power output (W), TTE (min) and $\dot{V}$O_2peak_ (l.min) from a continuously graded test on a cycle ergometer | MC phase does not affect peak exercise  capacity, with no changes in power output, TTE and $\dot{V}$O_2peak_ | Moderate |
| Davis *et al.* (1991) [15] | To determine whether MC phase affects muscle performance | Healthy females (n = 12) | EF, ovulation and ML | No information | Handgrip strength (N)  Standing long jump performance x body mass (kg.m) | Handgrip strength and performance was superior during the EF than both the ovulatory and ML phases of the MC. Standing long jump performance was again superior during the EF phase, although not with respect to the ML phase of the MC | Very low |
| Dean *et al.* (2003) [16] | To determine whether MC phase affects lactate threshold | Habitually active females (n = 8) | EF, LF and ML | MC history, counting of days, BBT and serum oestrogen and progesterone | $\dot{V}$O_2max_ and TTE (min) from an incremental graded-exercise test until volitional exhaustion on a cycle ergometer | There were no MC phase differences in $\dot{V}$O_2max_ and TTE | Low |
| De Bruyn-Prevost *et al.* (1984) [17] | To determine whether MC phase affects the physiological response to aerobic and anaerobic tests by young women | Healthy females (n = 7) | EF, ovulation and LL | BBT | Progressively increasing protocol for nine minutes on a cycle ergometer to determine $\dot{V}$O_2max_ (l.min^-1^), working capacity (W) and maximal pedalling time (s) | There were no differences in any performance variables measures across the MC | Very low |
| De Souza *et al.* (1990) [18] | To determine whether MC phase affects the physiological and metabolic responses to maximal and submaximal exercise in eumenorrheic runners | Well-conditioned female athletes (n = 8) | EF and ML | MC history, counting of days, urinary ovulation detection test (one month prior, during and one month post) and serum oestrogen and progesterone | Maximal exercise treadmill protocol to determine $\dot{V}$O_2max_ (ml.kg^-1^.min^-1^) and TTE (min) | No differences were observed for $\dot{V}$O_2max_ and TTE between MC phases | High |
| Dibrezzo *et al.* (1988) [19] | To determine whether MC phase affects dynamic strength and work performance of the knee flexors and extensors | Healthy females (n = 21) | EF, ovulation and LL | MC history, counting of days | Mean peak torque of knee flexors and extensors at 60, 180, 240º.s (Nm)  Muscular endurance and work ratios of knee flexors and extensors | There were no differences in mean peak torque or work ratios among the three MC phases | Very low |
| Dombovy *et al.* (1987) [20] | To determine whether MC phase affects the ventilatory response and exercise performance in normally menstruating, non-athletic women | Females not currently in active physical training (n = 8) | LF and ML | MC history, counting of days and serum oestrogen and progesterone | Incremental test on a cycle ergometer to determine $\dot{V}$O_2max_ (ml.kg^-1^.min^-1^) | There were no differences in $\dot{V}$O_2max_ across the MC | Moderate |
| Doolittle and Engebretsen (1972) [21] | To determine whether MC phase affects variations in performance | Healthy females (n = 16) | LF, ovulation, EL and LL | Counting of days | $\dot{V}$O_2max_ (ml.kg^-1^.min^-1^)  TTE during a 1.5-mile run-walk (s)  TTE during a 600-yard run-walk (s)  Distance covered during a 12-minute run-walk (miles) | There were no differences in any performance variable measured across the MC | Very low |
| Drake *et al.* (2003) [22] | To determine whether MC phase affects electromyography and mechanomyography during isometric muscle actions of the rectus femoris | Females not involved in any exercise program (n = 7) | EF, LF, ovulation and EL | MC history, counting of days, urinary ovulation detection test | Maximal torque during an isometric muscle action (Nm)  Torque at a sub-maximal (20, 50, 75%) isometric muscle action (Nm) | There were no differences in maximal and sub-maximal torque variables between MC phases | Low |
| Ekenros *et al.* (2013) [23] | To determine whether MC phase affects muscle strength in the upper and lower limb, as well as hop performance | Females involved in recreational physical activity (n = 9) | EF, ovulation and ML | Counting of days, urinary ovulation detection test and serum oestrogen and progesterone | Peak isokinetic muscle torque of knee extensors at 120º.s (Nm)  Handgrip strength (kg)  Peak length of hop during a one-leg hop test (cm) | No differences in handgrip strength and  hop performance were reported between MC phases. Peak torque of knee extensors changed across the MC whereby performance was greater in the ML phase compared to the EF phase | Moderate |
| ^a^Elliott *et al.* (2003) [24] | To determine whether MC phase affects maximum force production in young women | Sedentary females (n = 7) | EF and ML | MC history, counting of days, urinary ovulation detection test and serum oestrogen and progesterone | Maximum voluntary isometric force of the first dorsal interosseous (N) | There were no differences in muscle strength between the EF and ML phases of the MC | Moderate |
| ^b^Elliott *et al.* (2005) [25] | To determine whether MC phase affects muscle strength and sex hormone bioavailability | Healthy females (n = 7) | EF and ML | MC history, counting of days, urinary ovulation detection test and serum oestrogen and progesterone | Maximum voluntary isometric force of the first dorsal interosseous (N) | There were no differences in muscle strength during  the MC | High |
| Ettinger *et al.* (1998) [26] | To determine whether MC phase affects reflex responses to static handgrip at 30% maximal voluntary contraction in women | Healthy females (n = 10) | EF and LF | Serum oestrogen and progesterone | Handgrip strength (kg) | There were no differences in handgrip MVC strength between MC phases | Low |
| Frandsen *et al.* (2020) [27] | To determine the influence of the MC on whole body peak fat oxidation rate during a graded exercise test | Recreationally active females (n = 19) | LF, ovulation and ML | MC history and serum oestrogen and progesterone | $\dot{V}$O_2max_ (ml.kg^-1^.min^-1^) during a graded exercise test on a cycle ergometer until exhaustion | There were no differences in $\dot{V}$O_2max_ between MC phases | Low |
| Friden *et al.* (2003) [28] | To determine whether MC phase affects muscle strength and muscle endurance | Physically active females (n = 10) | EF, ovulation and ML | Counting of days, urinary ovulation detection test and serum oestrogen and progesterone | Peak handgrip strength (kg)  Best jump during a one-leg hop test (cm)  Maximal isokinetic muscle torque at 120º.s (Nm) | No variation in any performance variable was detected during the different phases of the MC | Low |
| Giacomoni *et al.* (2000) [29] | To determine whether MC phase affects maximal anaerobic performance during short-term anaerobic tests | Healthy females (n = 7) | EF, LF and ML | MC history, counting of days and serum progesterone | Maximal cycling power (W), optimal velocity (rpm) and optimal force (kg) during a force-velocity test  Maximal jump power during a multi-jump test (W.kg)  Maximal jump height from a squat jump test (cm) | No differences were observed in the force-velocity test or jump test performance among the three phases of the MC | Moderate |
| Girija and Veeraiah (2011) [30] | To determine whether MC phase affects physical working capacity in an Indian population | Healthy females (n = 40) | EF, LF and ML | Counting of days, serial follicular scanning | PWC (kg.m.min) performed on a cycle ergometer | PWC performance decreased in the ML and EF phases of the MC when compared to the LF phase | Very low |
| ^a^Gordon *et al.* (2012) [31] | To determine the effects of MC phase on the development of peak torque across a range of isokinetic speeds | Well trained female participants (n = 11) | EF, LF, ML and LL | MC history, counting of days, salivary oestrogen and progesterone | Torque production (Nm) of the knee extensors and flexors at 60, 80, 120 and 240º.s | There are fluctuations in peak torque of the knee extensors in response to phases of the MC | Very low |
| ^b^Gordon *et al.* (2017) [32] | To determine whether MC phase affects maximal oxygen uptake and associated cardio dynamic response | Physically active females (n = 10) | EF, LF, ML and LL | MC history, counting of days and salivary oestrogen and progesterone | Incremental stress test on a cycle ergometer to determine $\dot{V}$O_2max_ (l.min^-1^), TTE (s) and maximum power output (W) | There were no differences in $\dot{V}$O_2max_, TTE and maximum power output across the MC phases | Very low |
| ^a^Grucza *et al.* (1993) [33] | To determine whether MC phase affects changes in the thermo-sensitivity of the thermoregulatory system in exercising women | Physically active females (n = 10) | LF and ML | MC history and BBT (one month prior and during) | A maximal test on a cycle ergometer to determine $\dot{V}$O_2max_ (ml.kg^-1^.min^-1^) | $\dot{V}$O_2max_ did not differ between the phases of the MC | Very low |
| ^b^Grucza *et al.* (2002) [34] | To determine whether MC phase affects cardiorespiratory responses to exercise | Physically active females (n = 10) | LF and ML | MC history and BBT (one month prior and during) | A maximal test on a cycle ergometer to determine $\dot{V}$O_2max_ (ml.kg^-1^.min^-1^) | $\dot{V}$O_2max_ was greater in the LF phase compared with the ML phase of the MC | Very low |
| Gur (1997) [35] | To determine whether MC phase affects reliability of concentric and eccentric isokinetic measurements and reciprocal moment ratios in knee muscles | Sedentary women (n = 16) | EF, LF and ML | MC history, counting of days and serum oestrogen and progesterone | Torque ratios (peak and total) during concentric and eccentric hamstring and quadriceps testing at 60 and 180º.s | Concentric and eccentric peak torques, and total works, and their reciprocal ratio was not different among the MC phases | Low |
| Hertel *et al.* (2006) [36] | To determine whether MC phase affects hamstring and quadriceps strength, knee joint position sense, postural control and knee joint laxity | Competitive soccer or stunt cheerleading female athletes (n = 14) | LF, ovulation and ML. | MC history, counting of days, urinary ovulation detection test (one month prior) and urinary oestrogen and progesterone | Peak torque of quadricep and hamstring flexors and extensors at 120º.s (Nm)  Hamstring:quadricep strength ratio | There were  no differences in the  measures of strength (peak torque of hamstrings and hamstring:quadricep ratio) across the MC | Low |
| Hoeger-Bement *et al.* (2009) [37] | To determine whether MC phase affects exercise-induced analgesia in young women after a fatiguing isometric contraction | Healthy females (n = 20) | LF and ML | MC history, counting of days and urinary ovulation detection test | Time to task failure during a sustained isometric fatiguing contraction at 25% of MVC (s) performed in the upper-body | There was no difference in time to task failure of the sustained 25% MVC between the phases of the MC | Low |
| Hoshi (1997) [38] | To determine whether MC phase affects muscular strength, grip strength and back lift strength | Healthy females (n = 14) | EF, LF, ovulation and ML | Counting of days and serum oestrogen and progesterone | Handgrip strength (kg)  Back lift strength (kg) | Handgrip strength was lower in the EF phase compared with all other MC phases. Back lift strength was lower in the EF phase compared with all other MC phases and was higher in the LF compared with ovulation and the ML phases of the MC | Low |
| ^a^Janse de Jonge *et al.* (2001) [39] | To determine whether MC phase affects skeletal muscle strength, fatigue and contractile properties | Healthy females (n = 15) | EF, LF and ML | Counting of days, BBT, assessment of symptoms and serum oestrogen and progesterone | Isometric quadricep strength (N) with electrical stimulation  Isokinetic strength of the quadricep flexors and extensors at 60˚.s^_1^ (Nm)  Handgrip strength (N) | No changes were found in any of the muscle function parameters throughout the  MC | Low |
| ^b^Janse de Jonge *et al.* (2012) [40] | To determine whether MC phase affects prolonged exercise performance in both temperate and hot, humid conditions | Recreationally active females (n = 8) | EF and ML | Counting of days, BBT and serum oestrogen and progesterone | TTE (min) during a prolonged exercise performance test on a cycle ergometer at 60% $\dot{V}$O_2max_ followed by an incremental exercise test until exhaustion | In temperate  conditions, no changes in prolonged exercise performance were found over the MC | Moderate |
| Jarvis *et al.* (2011) [41] | To determine whether MC phase affects the cardiovascular and vasomotor sympathetic response during static handgrip to fatigue and post exercise circulatory arrest | Healthy females (n = 11) | EF and ML | Counting of days, urinary ovulation detection test and serum oestrogen and progesterone | Handgrip strength (kg)  Time to fatigue during static handgrip at 40% of MVC (s) | MC phase did not  influence MVC or time to fatigue | Moderate |
| Julian *et al.* (2017) [42] | To determine whether MC phase affects performance in soccer specific tests | High-level female soccer players (n = 9) | EF and ML | MC history, counting of days and serum oestrogen and progesterone | Sprint time (s) at 5, 10 and 30 m  CMJ height (cm)  Distance covered during Yo-Yo IET (m) | Yo-Yo IET performance was considerably lower during the ML phase as compared to the EF phase of the MC. There were no differences across the MC in all other performance variables | Low |
| Jurkowski *et al.* (1981) [43] | To determine whether MC phase affects exercise performance and the responses of oxygen transport, cardiac output and lactate production at several work rates | Healthy females (n = 9) | LF and ML | Counting of days, BBT and serum progesterone | Maximum power output (kpm.min) during a progressive incremental exercise test to exhaustion  on a cycle ergometer  Cycling TTE at 90% W_max_ (minutes) | There was no difference in maximum power output across the MC. TTE was greater during the ML phase compared to the LF phase of the MC | Low |
| Kaygisiz *et al.* (2003) [44] | To determine whether MC phase affects cardiorespiratory responses to exercise | Untrained females (n = 9) | LF and ML | MC history, counting of days and serum oestrogen and progesterone | Exercise test to exhaustion on a cycle ergometer determine $\dot{V}$O_2max_ (ml.kg^-1^.min^-1^) | MC phase did not affect $\dot{V}$O_2max_ | Low |
| Kraemer *et al.* (2006) [45] | To determine whether MC phase affects plasma proenkephalin peptide F responses to high intensity exercise in young untrained eumenorrheic women | Active females not participating in a regular training program (n = 8) | EF and ML | MC history, BBT and serum oestrogen and progesterone | TTE (s) during a progressive maximal exercise test performed on a cycle ergometer | There were no differences in exercise duration  between follicular and luteal phases | Low |
| Kubo *et al.* (2009) [46] | To determine whether MC phase affects changes in the mechanical properties of human muscle and tendon during the MC in vivo | Sedentary, or mildly to moderately active women (n = 8) | EF, ovulation and ML | MC history, BBT (two months prior and during) and serum oestrogen and progesterone | Maximal voluntary isometric strength of knee extensors and plantar flexors (Nm) with electrical stimulation | No change in muscle strength was found  during the MC | Moderate |
| ^a^Lara *et al.* (2019) [47] | To determine the effects of caffeine intake on  Wingate anaerobic test performance during three phases of the MC | Female triathletes (n = 13) | EF, ovulation and ML | MC history, BBT and urinary ovulation detection test | Peak power (W.kg), mean power (W.kg) and fatigue index (%) from a Wingate Test | There was no difference in Wingate test performance between MC phases | Low |
| ^b^Lara *et al.* (2019) [48] | To determine the ergogenic effects of caffeine in three phases of the MC | Female triathletes (n = 13) | EF, ovulation and ML | MC history, BBT and urinary ovulation detection test | Peak cycling power (W.kg) during a ramp test on a cycle ergometer until exhaustion | There was no difference in peak cycling power between MC phases | Low |
| Lebrun *et al.* (1995) [49] | To determine whether MC phase affects four selected induces of athletic performance: aerobic capacity, anaerobic capacity, isokinetic strength and high intensity endurance | Trained female athletes (n = 16) | EF and ML | MC history, ovulatory and menstrual symptoms, BBT and serum oestrogen and progesterone | $\dot{V}$O_2max_ (l.min) from a continuous progressive test until exhaustion on a treadmill  Anaerobic speed test (s)  TTE during an endurance run at 90% $\dot{V}$O_2max_ (s)  Quadriceps and hamstring strength at 30º.s (Nm) | A higher $\dot{V}$O_2max_ was reported in the EF phase compared to the ML phase. Anaerobic speed test, TTE and muscle strength was not influenced by MC phase | Low |
| Lee *et al.* (2014) [50] | To determine whether MC phase affects anterior cruciate ligament elasticity, force to flex the knee, and knee flexion–extension | Nonathletic females (n = 10) | EF, LF, ovulation and ML | MC history, counting of days and serum oestrogen and progesterone | Force to flex the knee from 90 to 125° (N) | Force to flex the knee was less at ovulation compared to the EF phase of the MC | Low |
| Lynch and Nimmo (1998) [51] | To determine whether MC phase affects intermittent exercise performance and some commonly used metabolic markers | Recreationally active females (n = 10) | LF and LL | MC history, counting of days and serum progesterone | TTE (s) during a 20 s repeat sprint continuous incremental protocol on a treadmill | There was no difference in performance between the LF and the LL phases of the MC | Moderate |
| Materson (1999) [52] | To determine whether MC phase affects anaerobic power performance | Fairly active females (n = 32) | EF and ML | MC history and counting of days | Anaerobic capacity (W), anaerobic power (W) and fatigue index (%) from a Wingate Test | Wingate performance improved in the ML phase compared with the EF phase of the MC | Very low |
| Mattu *et al.* (2019) [53] | To determine whether MC phase affects submaximal and maximal responses to exercise | Active females (n = 15) | LF and ML | MC history and urinary ovulation detection test | TTE on a cycle ergometer (s)  $\dot{V}$O_2max_ (l.min) and peak power output (W) from an incremental exercise test on a cycle ergometer | MC phase did not affect the submaximal and maximal exercise responses | Low |
| McCracken *et al.* (1994) [54] | To determine whether MC phase affects the blood lactate levels in response to intensive running | Physically active females (n = 9) | LF and ML | MC history, BBT (two months prior) and urinary oestrogen and progesterone | TTE (min) during an incremental and continuous exercise protocol on a treadmill | Running TTE was not different between the LF and ML phases of the MC | Low |
| McLay *et al.* (2007) [55] | To determine whether MC phase affects muscle-glycogen storage, exercise performance, and substrate metabolism at varying exercise intensities | Moderately trained women (n = 8) | LF and LL | BBT and serum oestrogen and progesterone | 16 km TT performance (min) on a cycle ergometer | TT performance was not affected by MC phase | Moderate |
| Montgomery and Shultz (2010) [56] | To determine whether MC phase affects maximal voluntary isometric contraction torque of the knee flexors and extensors | Recreationally active females (n = 29) | EF and EL | MC history, urinary ovulation detection kit and serum oestrogen and progesterone | Maximal voluntary isometric strength of the quadriceps and hamstrings (Nm.kg) | There was no difference in muscle strength between MC phases | Moderate |
| Okudan *et al.* (2005) [57] | To determine whether MC phase affects anaerobic performance | Sedentary females (n = 15) | LF, ovulation and ML | Serum oestrogen and progesterone | Anaerobic capacity (W), anaerobic power (W) and fatigue index (%) from a Wingate Test | There was no difference between the peak power,  mean power and fatigue index calculated in three different phases of the MC | Low |
| Oosthuyse *et al.* (2005) [58] | To determine whether MC phase affects exercise performance by means of a cycling time trial | Trained (n = 5) and untrained (n = 8) female cyclists | EF, LF and ML | Counting of days, BBT, urinary ovulation detection test and serum oestrogen and progesterone | 15 km TT performance (min) on a cycle ergometer  30 km TT performance (min) on a cycle ergometer | There was no difference in TT performance between MC phases in either the trained and untrained groups. Analysis of the combined trained and untrained group data revealed a trend for a faster TT time in the LF phase compared to the EF phase of the MC | High |
| Otaka *et al.* (2018) [59] | To determine whether MC phase affects tennis performance with and without dehydroepiandrosterone sulphate supplementation | Division 1 collegiate female tennis players (n = 10) | EF, LF, ovulation and ML | Counting of days | Isometric hip strength (Nm)  Tennis serve performance accuracy  Tennis serve performance velocity (mph) | The lowest tennis serve performance  score (attributed to a change in accuracy and not velocity) occurred at ovulation.  Isometric hip  strength decreased at ovulation | Very low |
| Pallavi *et al.* (2017) [60] | To determine whether MC phase affects muscle strength variations and also the rate of fatigue | Untrained or moderately trained female students (n = 100) | EF, LF and ML | MC history | Work done (J) during Mosso’s Ergograph test  Handgrip strength (kg) | The amount of work done and handgrip strength was higher in the LF phase and reduced in the EF and ML phases of the MC | Very low |
| Petrofsky *et al.* (2007) [61] | To determine whether MC phase affects isometric endurance and skin and muscle blood flow during isometric exercise for contractions at low, medium and high isometric tensions | Females not engaged in athletic programs (n = 8) | EF, LF, ovulation, EL, ML and LL | MC history | Endurance time at 20, 40 and 60% handgrip MVC (s) | There was small variation in endurance time across the MC for contractions at 60% handgrip MVC. This effect increased at 40% and was greatest at 20% handgrip MVC | Very low |
| Quadagno *et al.* (1991) [62] | To determine whether MC phase affects athletic performance as measured by weight lifting and swimming | Recreational female weight lifters (n = 12) and highly trained female swimmers (n = 15) | EF, LF and LL | MC history and counting of days | Bench and leg press mean weight lifted (pounds)  100-m freestyle swim time (s)  200-m freestyle swim time (s) | There were no differences in strength and swimming performance during the three MC phases | Very low |
| Redman *et al.* (2003) [63] | To determine whether MC phase affects the metabolic response to exercise | Sedentary females (n = 14) | LF and.ML | MC history, counting of days, urinary ovulation detection test and serum oestrogen and progesterone | Peak power output (W), TTE (min), total work done (kJ) and $\dot{V}$O_2max_ (l.min) from an incremental exercise test on a cycle ergometer | Incremental exercise test performance ($\dot{V}$O_2max_), TTE, maximal power output and total work done were not different between the two MC phases | High |
| Rodrigues *et al.* (2019) [64] | To determine whether MC affects MVC of lower limbs | Recreationally trained females (n = 12) | LL, EF and LF | Counting of days | MVC (kg) during leg press exercise | MVC was greater in the EF phase than the LL phase. MVC was greater in the LF phase then both the EF and LL phases. | Low |
| Romero-Moraleda *et al.* (2019) [65] | To determine whether MC phase affects muscle performance during half-squat exercise | Female triathletes (n = 13) | EF, ovulation and ML | MC history, BBT and urinary ovulation detection test | Mean and peak force at 20, 40, 60 and  80% of one-repetition maximum performed on a Smith Machine | Power outputs were very similar in all MC phases | Moderate |
| Sarwar *et al.* (1996) [66] | To determine whether MC phase affects skeletal muscle strength, contractile properties and fatiguability in young, healthy females | Relatively sedentary (n = 10) | EF, LF, ovulation, ML and LL | Counting of days | Maximum voluntary isometric strength of the quadriceps (N)  Handgrip strength (N) | There was an increase in  quadriceps and handgrip strength at ovulation compared with other MC phases | Very low |
| Shaharudin *et al.* (2011) [67] | To determine whether MC phase affects anaerobic capacity in repeated sprint cycling bouts | Moderately physically active females (n = 12) | LF and ML | BBT (three months prior and during), MC history and serum progesterone | Sprint duration until exhaustion throughout maximum accumulated oxygen deficit tests on a cycle ergometer | There were no differences between MC phases in sprint duration until exhaustion throughout maximum accumulated oxygen deficit tests | Low |
| Sipaviciene *et al.* (2013) [68] | To determine whether MC phase affects susceptibility to exercise-induced muscle damage after stretch-shortening cycle exercise | Physically active women (n = 18) | EF and ovulation | BBT and serum oestrogen and progesterone | Jump height (cm) from a drop jump  Maximum isometric torque of knee extensor muscles (Nm) with electrical stimulation | Jump height and MVC did not differ between MC phases | Low |
| Smekal *et al.* (2007) [69] | To determine whether MC phase affects the metabolic and cardiorespiratory responses to exercise | Active females (n = 19) | LF and LL | MC history, BBT and serum oestrogen | Power relative (W.kg) and $\dot{V}$O_2max_ (ml.min) from an incremental test until voluntary exhaustion on a cycle ergometer | There were no differences in power relative and $\dot{V}$O_2max_ across the MC | Low |
| ^a^Sunderland and Nevill (2003) [70] | To determine whether MC phase affects performance of high intensity intermittent running in the heat | Well trained female game players (n = 7) | LF and ML | Counting of days and serum oestrogen and progesterone | Distance ran during Loughborough Intermittent Shuttle Test (m)  15 m sprint time (s) | There were no differences in distance run or 15 m sprint time between MC phases | Low |
| ^b^Sunderland *et al.* (2011) [71] | To determine whether MC phase affects the growth hormone response to sprint exercise among normally menstruating women | Physically active females (n = 8) | LF and ML | Urinary ovulation detection test and serum oestrogen and progesterone | Mean and peak power outputs during an all-out 30 second sprint (W) on a treadmill | Mean and peak power outputs during an all-out 30 second sprint did not differ across the MC | Moderate |
| Takase *et al.* (2002) [72] | To determine whether MC phase affects induced modulations in the cardiorespiratory response to exercise with and without acute exposure to altitude | Moderately trained female athletes (n = 9) | LF and ML | MC history, BBT and serum oestrogen and progesterone | TTE (min) from an incremental maximal exercise test on a cycle ergometer | MC phase did not affect exercise TTE | Moderate |
| Tenan *et al.* (2016) [73] | To determine whether MC phase affects maximal isometric force and tremor during an endurance task | Recreationally active females (n = 9) | EF, LF, ovulation, ML and LL | MC history and BBT (one month prior) | MVC (Nm)  Mean time to task failure (s) during an endurance task | MVC in the ML phase was  lower than LF, ovulatory, and LL phases. There was no effect of MC phase on mean time to task failure | Very low |
| Tounsi *et al.* (2018) [74] | To determine whether MC phase affects soccer-related physical performance | Tunisian high-level soccer players (n = 11) | EF, LF and ML | Counting of days and serum progesterone | Distance covered during Yo-Yo IET (m)  Repeated shuttle-sprint ability test mean time (s)  Absolute performance during five-jump test (m) | None of the measured variables were altered due to MC phase | Low |
| Tsampoukos *et al.* (2010) [75] | To determine whether MC phase affects sprinting, recovery from sprinting and metabolic responses to sprinting | Highly active females (n = 8) | EF, ovulation and ML | MC history, urinary ovulation detection test and serum oestrogen and progesterone | Peak and mean power output during repeat sprint tests (W)  Fatigue index for power during repeat sprint tests (%)  Peak and mean speed during repeat sprint tests (m.s)  Fatigue index for speed during repeat sprint tests (m.s) | All performance variables were unaltered due to MC phase. | High |
| Vaiksaar *et al.* (2011) [76] | To determine whether MC phase affects endurance performance in trained rowers | Competitive female rowers (n = 8) | LF and ML | MC history, counting of days and serum oestrogen and progesterone | Incremental rowing ergometer test to determine $\dot{V}$O_2max_ (l.min) and maximal power output (W) | There were no differences in $\dot{V}$O_2max_ and power output between the two MC phases | Moderate |
| Wearing *et al.* (1972) [77] | To determine whether MC phase affects selected tests of physical fitness. | Female intercollegiate basketball or volleyball players | EF, LF, EL and LL | No information | Standing broad jump distance (inches)  Maximum hip flexion and extension strength (pounds) | Both standing broad jump and strength performance was reduced in the EF phase greatest in the LL phase of the MC | Very low |
| Wiecek *et al.* (2016) [78] | To determine whether MC phase affects the values of starting speed and anaerobic endurance | Physically active females (n = 16) | LF and ML | Counting of days, BBT and serum oestrogen and progesterone | Peak and mean power (W), time of attaining anaerobic peak power (s), time of maintaining anaerobic peak power (s) and power decrease (W.kg.s) during a maximal cycling sprint test | There were no differences between MC phases in the measured performance variables | Low |

BBT, basal body temperature; CMJ, counter-movement jump; EF, early follicular; EL, early luteal; IET, intermittent endurance test; LF, late follicular; LL, late luteal; MC, menstrual cycle; MILS, maximal isometric lifting strength; ML, mid-luteal; MVC, maximal voluntary contraction; PWC, physical working capacity; TTE, time to exhaustion; TT, time trial; $\dot{V}$O_2max,_ maximal oxygen uptake; $\dot{V}$O_2peak,_ peak oxygen uptake; v$\dot{V}$O_2max,_ velocity at maximal oxygen uptake.

*References*

1. Abt JP, Sell TC, Laudner KG, McCrory JL, Loucks TL, Berga SL, et al. Neuromuscular and biomechanical characteristics do not vary across the menstrual cycle. Knee Surg Sports Traumatolo Arthrosc. 2007;15(7):901-7.<https://doi.org/10.1007/s00167-007-0302-3>
2. Ansdell P, Brownstein CG, Škarabot J, Hicks KM, Simoes DC, Thomas K, et al. Menstrual cycle-associated modulations in neuromuscular function and fatigability of the knee extensors in eumenorrheic women. J Appl Physiol. 2019; 126(6):1701-12. <https://doi.org/10.1152/japplphysiol.01041.2018>
3. Bailey SP, Zacher CM, Mittleman KD. Effect of menstrual cycle phase on carbohydrate supplementation during prolonged exercise to fatigue. J Appl Physiol. 2000;88(2):690-7. <https://doi.org/10.1152/jappl.2000.88.2.690>
4. Bambaeichi E, Reilly T, Cable NT, Giacomoni M. The isolated and combined effects of menstrual cycle phase and time-of-day on muscle strength of eumenorrheic females. Chronobiol Int. 2004;21(4-5):645-60. <https://doi.org/10.1081/CBI-120039206>
5. Bandyopadhyay A, Dalui R. Endurance capacity and cardiorespiratory responses in sedentary females during different phases of menstrual cycle. **Kathmandu** Univ Med J. 2012;10(4):25-9. [https://doi.org/10.3126/kumj.v10i4.10990](https://doi.org/10.3126/kumj.v10i4.10990%20%20)
6. Beidleman BA, Rock PB, Muza SR, Fulco CS, Forte Jr VA, Cymerman A. Exercise V̇e and physical performance at altitude are not affected by menstrual cycle phase. J Appl Physiol. 1999;86(5):1519-26. <https://doi.org/10.1152/jappl.1999.86.5.1519>
7. Bell DR, Blackburn JT, Ondrak KS, Hackney AC, Hudson JD, Norcross MF, et al. The effects of oral contraceptive use on muscle stiffness across the menstrual cycle. Clin J Sport Med. 2011;21(6):467-73. <https://doi.org/10.1097/JSM.0b013e318230f50a>
8. Bemben DA, Salm PC, Salm AJ. Ventilatory and blood lactate responses to maximal treadmill exercise during the menstrual cycle. J Sports Med Phys Fitness. 1995;35(4):257-62.
9. ^a^Birch KM, Reilly T. Manual handling performance: the effects of menstrual cycle phase. Ergonomics. 1999;42(10):1317-32.
10. ^b^Birch K, Reilly T. The diurnal rhythm in isometric muscular performance differs with eumenorrheic menstrual cycle phase. Chronobiol Int. 2002;19(4):731-42. <https://doi.org/10.1081/CBI-120006083>
11. Burrows M, Bird SR. Velocity at $\dot{V}$O_2max_ and peak treadmill velocity are not influenced within or across the phases of the menstrual cycle. Euro J Appl Physiol. 2005;93(5-6):575-80. <https://doi.org/10.1007/s00421-004-1272-5>
12. Bushman B, Masterson G, Nelsen J. Anaerobic power performance and the menstrual cycle: eumenorrheic and oral contraceptive users. J Sports Med Phys Fitness. 2006;46(1):132.
13. Campbell SE, Angus DJ, Febbraio MA. Glucose kinetics and exercise performance during phases of the menstrual cycle: effect of glucose ingestion. Am J Physiol Endocrinol Metab. 2001;281(4):817-25. <https://doi.org/10.1152/ajpendo.2001.281.4.E817>
14. Casazza GA, Suh SH, Miller BF, Navazio FM, Brooks GA. Effects of oral contraceptives on peak exercise capacity. J Appl Physiol. 2002;93(5):1698-702. <https://doi.org/10.1152/japplphysiol.00622.2002>
15. Davies BN, Elford JC, Jamieson KF. Variations in performance in simple muscle tests at different phases of the menstrual cycle. J Sports Med Phys Fitness. 1991;31(4):532-7.
16. Dean TM, Perreault L, Mazzeo RS, Horton TJ. No effect of menstrual cycle phase on lactate threshold. J Appl Physiol. 2003;95(6):2537-43. <https://doi.org/10.1152/japplphysiol.00672.2003>
17. de Bruyn-Prevost P, Masset C, Sturbois X. Physiological response from 18-25 years women to aerobic and anaerobic physical fitness tests at different periods during the menstrual cycle. J Sports Med. 1984;24(2):144-8.
18. de Souza MS, Maguire MS, Rubin KR, Maresh CM. Effects of menstrual phase and amenorrhea on exercise performance in runners. Med Sci Sports Exerc. 1990;22(5):575-80. <https://doi.org/10.1249/00005768-199010000-00006>
19. Dibrezzo RO, Fort IL, Brown B. Dynamic strength and work variations during three stages of the menstrual cycle. J Orthop Sports Phys Ther. 1988;10(4):113-6.
20. Dombovy ML, Bonekat HW, Williams TJ, Staats BA. Exercise performance and ventilatory response in the menstrual cycle. Med Sci Sports Exerc. 1987;19(2):111-7.
21. Doolittle TL, Engebretsen J. Performance variations during the menstrual cycle. J Sports Med Phys Fitness. 1972;12(1):54.
22. Drake SM, Evetovich T, Eschbach C, Webster M. A pilot study on the effect of oral contraceptives on electromyography and mechanomyography during isometric muscle actions. J Electromyogr Kinesiol. 2003;13(3):297-301. <https://doi.org/10.1016/S1050-6411(03)00024-5>
23. Ekenros L, Hirschberg AL, Heijne A, Fridén C. Oral contraceptives do not affect muscle strength and hop performance in active women. Clin J Sport Med. 2013;23(3):202-7.<https://doi.org/10.1097/JSM.0b013e3182625a51>
24. ^a^Elliott KJ, Cable NT, Reilly T, Diver MJ. Effect of menstrual cycle phase on the concentration of bioavailable 17-β oestradiol and testosterone and muscle strength. Clin Sci. 2003;105(6):663-9. <https://doi.org/10.1042/CS20020360>
25. ^b^Elliott KJ, Cable NT, Reilly T. Does oral contraceptive use affect maximum force production in women?. Br J Sports Med. 2005;39(1):15-9. <http://dx.doi.org/10.1136/bjsm.2003.009886>
26. Ettinger SM, Silber DH, Gray KS, Smith MB, Yang QX, Kunselman AR, et al. Effects of the ovarian cycle on sympathetic neural outflow during static exercise. J Appl Physiol. 1998;85(6):2075-81.
27. Frandsen J, Pistoljevic N, Quesada JP, Amaro-Gahete FJ, Ritz C, Larsen S, et al. Menstrual cycle phase does not affect whole body peak fat oxidation rate during a graded exercise test. J Appl Physiol. 2020;128(3):681-7. <https://doi.org/10.1152/japplphysiol.00774.2019>
28. Fridén C, Hirschberg AL, Saartok T. Muscle strength and endurance do not significantly vary across 3 phases of the menstrual cycle in moderately active premenopausal women. Clin J Sport Med. 2003;13(4):238-41.
29. Giacomoni M, Bernard T, Gavarry O, Altare S, Falgairette G. Influence of the menstrual cycle phase and menstrual symptoms on maximal anaerobic performance. Med Sci Sports Exerc. 2000;32(2):486.
30. Girija B, Veeraiah SH. Effect of different phases of menstrual cycle on physical working capacity in Indian population. Indian J Physiol Pharmacol. 2011;55(2):165-9.
31. ^a^Gordon D, Hughes F, Young K, Scruton A, Keiller D, Caddy O, et al. The effects of menstrual cycle phase on the development of peak torque under isokinetic conditions. Isokinet Exerc Sci. 2013;21(4):285-91.
32. ^b^Gordon D, Scruton A, Barnes R, Baker J, Prado L, Merzbach V. The effects of menstrual cycle phase on the incidence of plateau at and associated cardiorespiratory dynamics. Clin Physiol Funct Imaging. 2018; 38(4):689-98. <https://doi.org/10.1111/cpf.12469>
33. ^a^Grucza R, Pekkarinen H, Titov EK, Kononoff A, Hänninen O. Influence of the menstrual cycle and oral contraceptives on thermoregulatory responses to exercise in young women. Eur J Appl Physiol Occup Physiol. 1993;67(3):279-85.
34. ^b^Grucza R, Pekkarinen H, Hanninen O. Cardiorespiratory responses to bicycle incremental exercise in women taking oral contraceptives. Biol Sport. 2002;19(3):267-79.
35. Gür H. Concentric and eccentric isokinetic measurements in knee muscles during the menstrual cycle: a special reference to reciprocal moment ratios. Arch Phys Med Rehabil. 1997;78(5):501-5.
36. Hertel J, Williams NI, Olmsted-Kramer LC, Leidy HJ, Putukian M. Neuromuscular performance and knee laxity do not change across the menstrual cycle in female athletes. Knee Surg Sports Traumatol Arthrosc. 2006;14(9):817-22. <https://doi.org/10.1007/s00167-006-0047-4>
37. Hoeger Bement MK, Rasiarmos RL, DiCapo JM, Lewis A, Keller ML, Harkins AL, et al. The role of the menstrual cycle phase in pain perception before and after an isometric fatiguing contraction. Eur J Appl Physiol. 2009;106(1):105-12.
38. Hoshi A. Changes in muscular strength of women in different phases of the menstrual cycle. Bulletin of the Nippon Dental University. 1997;26:219-24.
39. ^a^Janse de Jonge X, Boot CR, Thom JM, Ruell PA, Thompson MW. The influence of menstrual cycle phase on skeletal muscle contractile characteristics in humans. J Physiol. 2001;530(1):161-6.<https://doi.org/10.1111/j.1469-7793.2001.0161m.x>
40. ^b^Janse de Jonge X, Thompson MW, Chuter VH, Silk LN, Thom JM. Exercise performance over the menstrual cycle in temperate and hot, humid conditions. Med Sci Sports Exerc. 2012;44(11):2190-8. <https://doi.org/10.1249/mss.0b013e3182656f13>
41. Jarvis SS, VanGundy TB, Galbreath MM, Shibata S, Okazaki K, Reelick MF, et al. Sex differences in the modulation of vasomotor sympathetic outflow during static handgrip exercise in healthy young humans. Am J Physiol Regul Integr Comp Physiol. 2011;301(1):193-200.
42. Julian R, Hecksteden A, Fullagar HH, Meyer T. The effects of menstrual cycle phase on physical performance in female soccer players. PloS one. 2017;12(3):e0173951. <https://doi.org/10.1371/journal.pone.0173951>
43. Jurkowski JE, Jones NL, Toews CJ, Sutton JR. Effects of menstrual cycle on blood lactate, O2 delivery, and performance during exercise. J Appl Physiol. 1981;51(6):1493-9.
44. Kaygisiz Z, Erkasap N, Soydan M. Cardiorespiratory responses to submaximal incremental exercise are not affected by one night's sleep deprivation during the follicular and luteal phases of the menstrual cycle. Indian J Pharmacol. 2003;47(3):279-87.
45. Kraemer WJ, Kim SK, Bush JA, Nindl BC, Volek JS, Spiering BA, et al. Influence of the menstrual cycle on proenkephalin peptide F responses to maximal cycle exercise. Euro J Appl Physiol. 2006;96(5):581-6.
46. Kubo K, Miyamoto M, Tanaka S, Maki A, Tsunoda N, Kanehisa H. Muscle and tendon properties during menstrual cycle. Int J Sports Med. 2009;30(2):139-43. <https://doi.org/10.1055/s-0028-1104573>
47. ^a^Lara B, Gutiérrez Hellín J, Ruíz‐Moreno C, Romero‐Moraleda B, Del Coso J. Acute caffeine intake increases performance in the 15‐s Wingate test during the menstrual cycle. Br J Clin. 2019;86:745-752. <https://doi.org/10.1111/bcp.14175>
48. ^b^Lara B, Gutiérrez-Hellín J, García-Bataller A, Rodríguez-Fernández P, Romero-Moraleda B, Del Coso J. Ergogenic effects of caffeine on peak aerobic cycling power during the menstrual cycle. Euro J Nutr. 2019;1-10. <https://doi.org/10.1007/s00394-019-02100-7>
49. Lebrun CM, McKenzie DC, Prior JC, Taunton JE. Effects of menstrual cycle phase on athletic performance. Med Sci Sports Exerc. 1995;27(3):437-44.
50. Lee H, Petrofsky JS, Daher N, Berk L, Laymon M. Differences in anterior cruciate ligament elasticity and force for knee flexion in women: oral contraceptive users versus non-oral contraceptive users. Euro J Appl Physiol. 2014;114(2):285-94. <https://doi.org/10.1007/s00421-013-2771-z>
51. Lynch NJ, Nimmo MA. Effects of menstrual cycle phase and oral contraceptive use on intermittent exercise. Eur J Appl Physiol Occup Physiol. 1998;78(6):565-72.
52. Masterson G. The impact of menstrual phases on anaerobic power performance in collegiate women. J Strength Cond Res. 1999;13(4):325-9.
53. Mattu AT, Iannetta D, MacInnis MJ, Doyle‐Baker PK, Murias JM. Menstrual and oral contraceptive cycle phases do not affect submaximal and maximal exercise responses. Scand J Med Sci Sports. 2019. <https://doi.org/10.1111/sms.13590>
54. McCracken M, Ainsworth B, Hackney AC. Effects of the menstrual cycle phase on the blood lactate responses to exercise. Eur J Appl Physiol Occup Physiol. 1994;69(2):174-5.
55. McLay RT, Thomson CD, Williams SM, Rehrer NJ. Carbohydrate loading and female endurance athletes: effect of menstrual-cycle phase. Int J Sport Nutr Exerc Metab. 2007;17(2):189-205. <https://doi.org/10.1123/ijsnem.17.2.189>
56. Montgomery MM, Shultz SJ. Isometric knee-extension and knee-flexion torque production during early follicular and postovulatory phases in recreationally active women. J Athl Train. 2010;45(6):586-93.
57. Okudan N, Gokbel H, Ucok K, Baltaci A. Serum leptin concentration and anaerobic performance do not change during the menstrual cycle of young females. Neuroendocrinology Letters. 2005;26(4):297-300.
58. Oosthuyse T, Bosch AN, Jackson S. Cycling time trial performance during different phases of the menstrual cycle. Euro J Appl Physiol. 2005;94(3):268-76. 10.1007/s00421-005-1324-5
59. Otaka M, Chen SM, Zhu Y, Tsai YS, Tseng CY, Fogt DL, et al. Does ovulation affect performance in tennis players?. Br J Sports Med. 2018;4(1):e000305. <http://dx.doi.org/10.1136/bmjsem-2017-000305>
60. Pallavi LC, SoUza UJ, Shivaprakash G. Assessment of musculoskeletal strength and levels of fatigue during different phases of menstrual cycle in young adults. J Clin Diagn Res. 2017;11(2):11-13.
61. Petrofsky J, Al Malty A, Suh HJ. Isometric endurance, body and skin temperature and limb and skin blood flow during the menstrual cycle. Med Sci Moni. 2007;13(3):111-7.
62. Quadagno D, Faquin L, Lim GN, Kuminka W, Moffatt R. The menstrual cycle: does it affect athletic performance?. Physician Sports Med. 1991;19(3):121-4.
63. Redman LM, Scroop GC, Norman RJ. Impact of menstrual cycle phase on the exercise status of young, sedentary women. Euro J Appl Physiol. 2003;90(5-6):505-13. <https://doi.org/10.1007/s00421-003-0889-0>
64. Rodrigues P, de Azevedo Correia M, Wharton L. Effect of menstrual cycle on muscle strength. J Exerc Physiol Online. 2019;22(5):89-96.
65. Romero-Moraleda B, Del Coso J, Gutiérrez-Hellín J, Ruiz-Moreno C, Grgic J, Lara B. The influence of the menstrual cycle on muscle strength and power performance. J Hum Kinet. 2019;68:123-133.
66. Sarwar R, Niclos BB, Rutherford OM. Changes in muscle strength, relaxation rate and fatiguability during the human menstrual cycle. J Physiol. 1996;493(1):267-72.
67. Shaharudin S, Ghosh AK, Ismail AA. Anaerobic capacity of physically active eumenorrheic females at mid-luteal and mid-follicular phases of ovarian cycle. J Sports Med Phys Fitness. 2011;51(4):576.
68. Sipavičienė S, Daniusevičiutė L, Klizienė I, Kamandulis S, Skurvydas A. Effects of estrogen fluctuation during the menstrual cycle on the response to stretch-shortening exercise in females. BioMed Res Int. 2013; 2013:1-6. <http://dx.doi.org/10.1155/2013/243572>
69. Smekal G, Von Duvillard SP, Frigo P, Tegelhofer T, Pokan R, Hofmann P, et al. Menstrual cycle: no effect on exercise cardiorespiratory variables or blood lactate concentration. Med Sci Sports Exerc. 2007;39(7):1098-106. <https://doi.org/10.1249/mss.0b013e31805371e7>
70. ^a^Sunderland C, Nevill M. Effect of the menstrual cycle on performance of intermittent, high-intensity shuttle running in a hot environment. Euro J Appl Physiol. 2003;88(4-5):345-52. <https://doi.org/10.1007/s00421-002-0722-1>
71. ^b^Sunderland C, Tunaley V, Horner F, Harmer D, Stokes KA. Menstrual cycle and oral contraceptives’ effects on growth hormone response to sprinting. Appl Physiol Nutr Metab. 2011;36(4):495-502. <https://doi.org/10.1139/h11-039>
72. Takase K, Nishiyasu T, Asano K. Modulating effects of the menstrual cycle on cardiorespiratory responses to exercise under acute hypobaric hypoxia. Japanese J Physiol. 2002;52(6):553-60. <https://doi.org/10.2170/jjphysiol.52.553>
73. Tenan MS, Hackney AC, Griffin L. Maximal force and tremor changes across the menstrual cycle. Eur J Appl Physiol. 2016;116(1):153-60. <https://doi.org/10.1007/s00421-015-3258-x>
74. Tounsi M, Jaafar H, Aloui A, Souissi N. Soccer-related performance in eumenorrheic Tunisian high-level soccer players: effects of menstrual cycle phase and moment of day. J Sports Med Phys Fitness. 2018;58(4):497-502. <https://doi.org/10.23736/s0022-4707.17.06958-4>
75. Tsampoukos A, Peckham EA, James R, Nevill ME. Effect of menstrual cycle phase on sprinting performance. Eur J Appl Physiol. 2010;109(4):659-67. <https://doi.org/10.1007/s00421-010-1384-z>
76. Vaiksaar S, Jürimäe J, Mäestu J, Purge P, Kalytka S, Shakhlina L, et al. No effect of menstrual cycle phase and oral contraceptive use on endurance performance in rowers. J Strength Cond Res. 2011;25(6):1571-8. <https://doi.org/10.1519/JSC.0b013e3181df7fd2>
77. Wearing MP, Yuhosz MD, Campbell R, Love EJ. The effect of the menstrual cycle on tests of physical fitness. J Sports Med Phys Fitness. 1972;12(1):38-41.
78. Wiecek M, Szymura J, Maciejczyk M, Cempla J, Szygula Z. Effect of sex and menstrual cycle in women on starting speed, anaerobic endurance and muscle power. Acta Physiologica Hungarica. 2016;103(1):127-32. <https://doi.org/10.1556/036.103.2016.1.13>
